# AlCl_3_-Catalyzed Cascade Reactions of 1,2,3-Trimethoxybenzene and Adipoyl Chloride: Spectroscopic Investigations and Density Functional Theory Studies

**DOI:** 10.1021/acsomega.2c04612

**Published:** 2022-10-14

**Authors:** Yasin Çetinkaya, Tekin Artunç, Abdullah Menzek

**Affiliations:** Department of Chemistry, Faculty of Science, Atatürk University, 25240Erzurum, Turkey

## Abstract

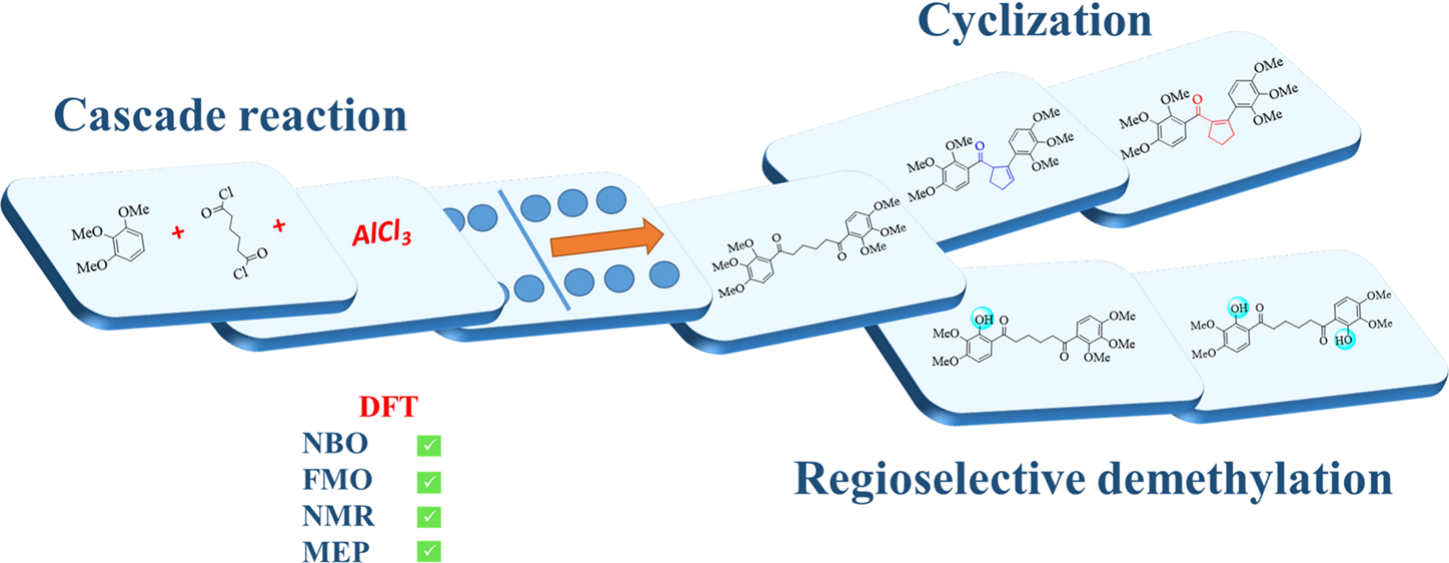

The reaction of 1,2,3-trimethoxybenzene with adipoyl chloride in the presence of AlCl_3_ gave two isomeric cyclopentene derivatives, 1,6-bis(2,3,4-trimethoxyphenyl)hexane-1,6-dione, and two demethylation products of aryl methyl ethers. The cyclopentene derivatives including unconjugated or conjugated enones are products formed in a cascade reaction resulting from first the Friedel–Crafts acylation reaction and then aldol condensation. All compounds were optimized by density functional theory calculated using two functional levels, B3LYP and M06-2X, with the 6-311+G(d,p) basis set. The structural properties were established, natural bond orbital analysis of donor–acceptor interactions was carried out, and charges on the atoms and quantum chemical reactivity identifiers were determined to compare the strength of the intramolecular hydrogen bonds formed and their stabilities. To compare the experimental ^1^H and ^13^C NMR chemical shifts with the calculated values, NMR chemical shift calculations were carried out using the gauge-invariant atomic orbital method.

## 1 Introduction

**Figure 1:**
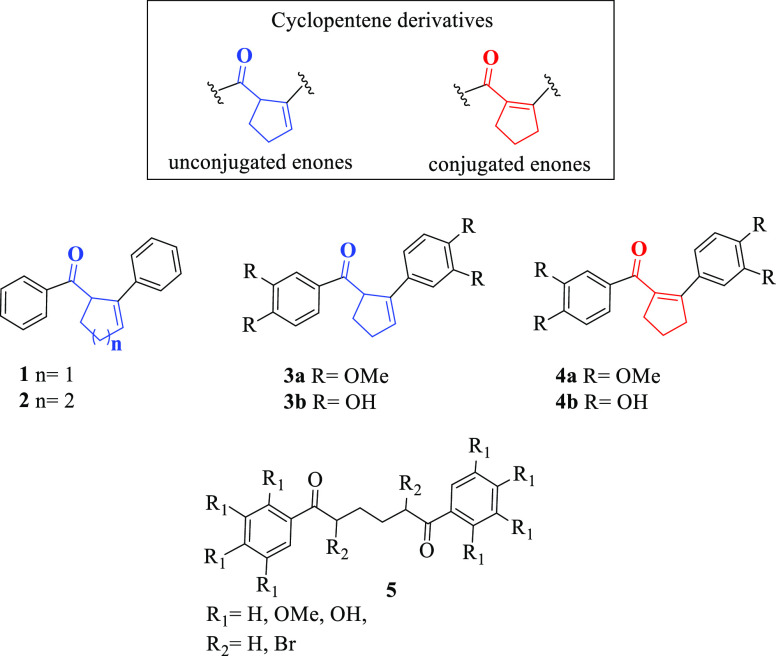
Some cyclopentene derivatives (**1**–**4**) and 1,6-diphenylhexane-1,6-dione derivatives (**5**).

Friedel–Crafts acylation^1,2^ and aldol condensation^3^ reactions are two methods widely used to achieve fundamental organic reactions for C–C bond formation. By using these methods, many natural products and derivatives with bromophenol structures have been synthesized.^4−9^ There are very few studies in the literature in which these two reactions occur consecutively. Miyahara and Ito described a new cyclization method to form five- and six-membered rings (**1** and **2**) under mild reaction conditions via these two reactions (Figure 1). They reported that unconjugated enones were obtained instead of the more thermodynamically stable conjugated enones obtained by base-catalyzed aldol condensation.^10^ They also stated in the references section of the article that in a study by Cǎlin and Frǎsineanu, the main product in base-catalyzed aldol condensation of 1,6-bis(2,5-dimethylphenyl)hexane-1,6-dione is an unconjugated cyclocondensation product.^10,11^ However, they also reported that they examined the ^1^H NMR spectrum of the article and reported that the product was a mixture, and only a small amount of the unconjugated product was formed. Kakemi et al. stated that they synthesized bis(2-hydroxy-3,4-dimethoxyphenyl)alkane dione derivatives by the Friedel–Crafts reaction using 1,2,3-trimethoxybenzene and dicarboxylic chloride derivatives. In that reaction, they only obtained the demethylation product of both aryl methyl ethers in the *ortho* positions, and then they converted these OH groups to the methoxy groups with dimethyl sulfate.^12^ On the other hand, unconjugated enones were formed by acid-catalyzed aldol condensation of 1,4-dibenzoylbutane, also called 1,6-diphenylhexane-1,6-dione.^10,13,14^

Very recently, by using these methods, we reported the synthesis of 1,6-diphenylhexane-1,6-dione derivatives (**5**), cyclopentenyl methanone derivatives **3a** and **4a**, and their hydroxylated derivatives **3b** and **4b** starting from 3,4-dimethoxybenzene and adipoyl chloride in the presence of AlCl_3_ (Figure 1).^15,16^ In this reaction, in which the main product was conjugated cyclocondensation product **3a**, substituted cyclopentene derivatives **3a** and **4a** were isolated in yields of 75 and 16%, respectively (Figure 1). Moreover, disubstituted cyclopentene and cyclohexene derivatives (with similar structures to **3a** and **4a**) from cyclocondensation reactions of di(thiophen-2-yl)alkane diones in HCl/HOAc were synthesized.^17^

In our previous work, we synthesized derivatives of compound **5** and investigated their biological activities. The derivatives of these phenolic compounds were observed to have antidiabetic potential in metabolic enzymes as well as important biological activities such as acetylcholinesterase and carbonic anhydrase (CA) inhibition, anti-inflammatory, and antioxidant.^18−29^ In two of these studies, regioselective demethylation of aryl methyl ethers using BBr_3_ or Br_2_ was observed.^21,22^ Regioselective demethylation in aryl methyl ethers can be achieved using Lewis or protic acids such as AlCl_3_, BBr_3_, BCl_3_, MgI_2_, BeCl_2_, HI, and HBr.^22,30−33^ We reported regioselective demethylation in aryl methyl ethers, which occurs with HBr formed in an electrophilic aromatic substitution reaction in the presence of Br_2_.^21^

Herein, in continuation of these studies, we decided to investigate in detail the reaction of 1,2,3-trimethoxybenzene and adipoyl chloride in the presence of AlCl_3_. Because *ortho* and *para* substituted aryl methyl ethers will be formed in this reaction, regioselective demethylation products can be formed by the cascade reaction in the presence of AlCl_3_, a Lewis acid. Natural bond orbital (NBO) analysis and molecular electrostatic potential (MEP) analysis were performed, the structural parameters of all compounds were determined, and the gauge-invariant atomic orbital (GIAO) approach for calculating the NMR chemical shifts was applied using density functional theory (DFT) calculations including two functional levels, M06-2X and B3LYP, with the 6-311+G(d,p) basis set.

## 2 Materials and Methods

### 2.1 Instrumentation and Chemicals

All reagents and solvents were used as purchased from their commercial provider without any purification. All column chromatography studies were performed on silica gel (60-mesh, Merck). Melting points were determined on a melting-point apparatus (Gallenkamp; WA11373) and are uncorrected. The ^1^H and ^13^C NMR spectra were recorded on Varian and Bruker spectrometers at 400 (^1^H) and 100 MHz (^13^C), and NMR shifts are presented as δ in ppm. The IR spectra were obtained from solutions in 0.1 mm cells with a PerkinElmer spectrophotometer. High-resolution mass spectrometry (HRMS) of all compounds was carried out using a quadrupole time-of-flight spectrometry device (1200/6210, Agilent).

### 2.2 Computational Details

DFT is an excellent computational method for obtaining vibrational frequencies, molecular interactions, and mechanical insights as well as thermodynamic and kinetic stability.^34−38^ This computational method is also used with hybrid functionals to calculate the structural, optical, and electronic properties of molecular systems and atoms.^39−41^ All computations were performed with the software package Gaussian 09W^42^ and carried out by DFT using B3LYP (Becke’s three parameter hybrid functional combined with the Lee–Yang–Parr correlation functional)^43,44^ and M06-2X^45,46^ hybrid functionals with the 6-311+G(d,p) basis set in the gas phase. The results were visualized using the software GaussView 5 and CYLview v1.0.561 BETA for data preparation and visualization of the results.^47^ The ^1^H and ^13^C NMR chemical shifts were calculated using the GIAO method^48,49^ based on the optimized geometries. The frontier molecular orbital (FMO) analysis, NBO calculations and MEP analysis were performed, and global chemical reactivity descriptors were calculated using DFT at the B3LYP/311G+(d,p) and M06-2X/311G+(d,p) level in the gas phase.

### 2.3 Synthesis

#### 2.3.1 Reaction of 1,2,3-Trimethoxybenzene (**6**) with Adipoyl Chloride (**7**) in the Presence of AlCl_3_

To a solution of 1,2,3-trimethoxybenzene (**6**) (5.0 g, 30 mmol) in CH_2_Cl_2_ (40 mL) were added adipoyl chloride (**7**) (2.723 g, 15 mmol) and AlCl_3_ (4.37 g, 33 mmol) at rt. It was observed by thin-layer chromatography (TLC) that the reaction was completed at the same temperature after 3 h. Water (50 mL) and CH_2_Cl_2_ (50 mL) were added to the reaction mixture, consecutively. After the organic phase was separated, the aqueous phase was extracted with CH_2_Cl_2_ (2 × 50 mL). After the combined organic phases were dried over Na_2_SO_4_, the solvent was removed in the evaporator. Five stains were observed by TLC of the dark red mixture (9.30 g). The residual mixture was chromatographed on a silica gel column (90 g) with EtOAc/hexane (9,1). Cyclopentene derivatives **8** (1.12 g, 9%) and **9** (1.35 g, 11%) and diketones **10** (1.39 g, 10%), **11** (0.97 g, 8%), and **12** (4.02 g, 32%) were isolated.

##### 2.3.1.1 (2,3,4-Trimethoxyphenyl)(2-(2,3,4-trimethoxyphenyl)cyclopent-2-en-1-yl)methanone (**8**)

Yellow oil, *R*_f_ (20% EtOAc/hexane) 0.24; ^1^H NMR (400 MHz, CDCl_3_) δ (ppm): 7.31 (d, A part of AB system, *J* = 8.7 Hz, 1H, aromatic), 6.89 (d, A part of AB system, *J* = 8.7 Hz, 1H, aromatic), 6.64 (d, B part of AB system, *J* = 8.7 Hz, 1H, aromatic), 6.53 (d, B part of AB system, *J* = 8.7 Hz, 1H, aromatic), 6.33 (bs, 1H, CH, olefinic), 5.04–4.97 (m, 1H, CH, aliphatic), 3.97 (s, 3H, OCH_3_), 3.88 (s, 3H, OCH_3_), 3.87 (s, 3H, OCH_3_), 3.81 (s, 3H, OCH_3_), 3.79 (s, 3H, OCH_3_), 3.76 (s, 3H, OCH_3_), 2.68–2.48 (m, 2H, CH_2_), 2.47–2.34 (m, 1H, CH_2_), 2.19–2.07 (m, 1H, CH_2_); ^13^C NMR (100 MHz, CDCl_3_) δ (ppm): 202.9 (CO), 156.8 (C), 153.5 (C), 152.6 (C), 151.6 (C), 142.3 (C), 141.9 (C), 139.4 (C), 131.8 (CH), 126.7 (C), 125.6 (CH), 123.8 (C), 123.6 (CH), 107.0 (CH), 106.9 (CH), 61.6 (OCH_3_), 60.82 (OCH_3_), 60.80 (OCH_3_), 60.3 (OCH_3_), 58.5 (OCH_3_), 56.0, 55.9, 32.2 (CH_2_), 29.5 (CH_2_). IR (CH_2_Cl_2_, cm^–1^): 3405, 2937, 2844, 1670, 1590, 1493, 1462, 1411, 1292, 1251, 1206, 1101, 1012, 932, 796, 694. HRMS (ESI-TOF) *m/z*: [M]^+^ calcd for C_24_H_28_O_7_: 428.1835; found: 428.1838.

##### 2.3.1.2 (2,3,4-Trimethoxyphenyl)(2-(2,3,4-trimethoxyphenyl)cyclopent-1-en-1-yl)methanone (**9**)

Yellow solid, Mp 95–97 °C; *R*_f_ (20% EtOAc/hexane) 0.21; ^1^H NMR (400 MHz, CDCl_3_) δ (ppm): 7.01 (d, A part of AB system, *J* = 8.6 Hz, 1H, aromatic), 6.68 (d, A part of AB system, *J* = 8.6 Hz, 1H, aromatic), 6.384 (d, B part of AB system, *J* = 8.6 Hz, 1H, aromatic), 6.376 (d, B part of AB system, *J* = 8.6 Hz, 1H, aromatic), 3.87 (s, 3H, OCH_3_), 3.79 (s, 3H, OCH_3_), 3.76 (s, 3H, OCH_3_), 3.74 (s, 3H, OCH_3_), 3.73 (s, 3H, OCH_3_), 3.71 (s, 3H, OCH_3_), 2.96–2.85 (m, 4H, CH_2_), 2.11–1.99 (m, 2H, CH_2_); ^13^C NMR (100 MHz, CDCl_3_) δ (ppm): 195.2 (CO), 155.6 (C), 153.4 (C), 152.6 (C), 151.0 (C), 148.6 (C), 141.78 (C), 141.76 (C), 140.4 (C), 128.3 (C), 125.4 (CH), 124.7 (CH), 124.3 (C), 106.6 (CH), 106.2 (CH), 62.0 (OCH_3_), 60.9 (OCH_3_), 60.85 (OCH_3_), 60.79 (OCH_3_), 56.1 (OCH_3_), 56.1 (OCH_3_), 40.2 (CH_2_), 35.4 (CH_2_), 22.9 (CH_2_). IR (CH_2_Cl_2_, cm^–1^): 2940, 2840, 1638, 1593, 1494, 1463, 1411, 1353, 1285, 1234, 1101, 1052, 1019, 982, 798, 751, 693. HRMS (ESI-TOF) *m/z*: [M + H]^+^ calcd for C_24_H_28_O_7_: 429.1869; found: 429.1909.

##### 2.3.1.3 1,6-Bis(2,3,4-trimethoxyphenyl)hexane-1,6-dione (**10**)

Colorless crystal, Mp 113–115 °C (Lit.^12^ 116 °C); *R*_f_ (20% EtOAc/hexane) 0.16; ^1^H NMR (400 MHz, CDCl_3_) δ (ppm): 7.43 (d, A part of AB system, *J* = 8.9 Hz, 2H, aromatic), 6.68 (d, B part of AB system, *J* = 8.9 Hz, 2H, aromatic), 3.93 (s, 6H, OCH_3_), 3.88 (s, 6H, OCH_3_), 3.84 (s, 6H, OCH_3_), 3.00–2.92 (m, 4H, CH_2_), 1.77–1.70 (m, 4H, CH_2_); ^13^C NMR (100 MHz, CDCl_3_) δ (ppm): 201.1 (CO), 157.3 (C), 154.0 (C), 142.2 (C), 126.4 (C), 125.5 (CH), 107.3 (CH), 61.6 (OCH_3_), 61.0 (OCH_3_), 56.3 (OCH_3_), 43.1 (CH_2_), 24.4 (CH_2_). IR (CH_2_Cl_2_, cm^–1^): 2950, 2914, 2845, 1663, 1587, 1493, 1467, 1411, 1369, 1288, 1268, 1218, 1199, 1172, 1096, 1028, 985, 921, 886, 804, 772, 729, 697, 563, 433.

##### 2.3.1.4 1-(2-Hydroxy-3,4-dimethoxyphenyl)-6-(2,3,4-trimethoxyphenyl)hexane-1,6-dione (**11**)

Colorless solid, Mp 58–60 °C; *R*_f_ (20% EtOAc/hexane) 0.12; ^1^H NMR (400 MHz, CDCl_3_) δ (ppm): 12.64 (s, 1H, OH), 7.51 (d, A part of AB system, *J* = 9.1 Hz, 1H, aromatic), 7.43 (d, A part of AB system, *J* = 8.8 Hz, 1H, aromatic), 6.68 (d, B part of AB system, *J* = 8.8 Hz, 1H, aromatic), 6.46 (d, B part of AB system, *J* = 9.1 Hz, 1H, aromatic), 3.93 (s, 3H, OCH_3_), 3.90 (s, 3H, OCH_3_), 3.87 (s, 3H, OCH_3_), 3.85 (s, 3H, OCH_3_), 3.84 (s, 3H, OCH_3_), 3.03–2.91 (m, 4H, CH_2_), 1.81–1.73 (m, 4H, CH_2_); ^13^C NMR (100 MHz, CDCl_3_) δ (ppm): 205.5 (CO), 200.7 (CO), 158.5 (C), 157.41 (C), 154.0 (C), 142.2 (C), 136.8 (C), 126.5 (CH), 126.2 (C), 125.5 (CH), 115.1 (C), 107.3 (CH), 103.1 (CH), 61.6 (OCH_3_), 61.0 (OCH_3_), 60.8 (OCH_3_), 56.3 (OCH_3_), 56.3 (OCH_3_), 42.9 (CH_2_), 38.2 (CH_2_), 24.6 (CH_2_), 24.3 (CH_2_). IR (CH_2_Cl_2_, cm^–1^): 2939, 1670, 1632, 1589, 1507, 1493, 1461, 1447, 1412, 1357, 1290, 1209, 1129, 1103, 1091, 1008, 792, 700. HRMS (ESI-TOF) *m/z*: [M]^+^ calcd for C_23_H_28_O_8_: 432.1784; found: 432.1779.

##### 2.3.1.5 1,6-Bis(2-hydroxy-3,4-dimethoxyphenyl)hexane-1,6-dione (**12**)

Light yellow solid, Mp 172–174 °C (Lit.^12^ 175 °C); *R*_f_ (20% EtOAc/hexane) 0.09; ^1^H NMR (400 MHz, CDCl_3_) δ (ppm): 12.62 (s, 2H, OH), 7.52 (d, A part of AB system, *J* = 9.1 Hz, 2H, aromatic), 6.48 (d, B part of AB system, *J* = 9.1 Hz, 2H, aromatic), 3.92 (s, 6H, OCH_3_), 3.87 (s, 6H, OCH_3_), 3.02–2.95 (m, 4H, CH_2_), 1.86–1.80 (m, 4H, CH_2_); ^13^C NMR (100 MHz, CDCl_3_) δ (ppm): 205.2 (CO), 158.7 (C), 157.4 (C), 136.9 (C), 126.4 (CH), 115.1 (C), 103.2 (CH), 60.9 (OCH_3_), 56.3 (OCH_3_), 38.0 (CH_2_), 24.4 (CH_2_). IR (CH_2_Cl_2_, cm^–1^): 2952, 1637, 1504, 1444, 1421, 1278, 1127, 1074, 990, 870, 784.

#### 2.3.2 Reaction of Diketone **12** with Bromine

After dissolving diketone **12** (0.1 g, 0.24 mmol) in CH_2_Cl_2_ (20 mL), Br_2_ (1 mL) was added dropwise over 5 min at room temperature. After the mixture was stirred at the same temperature for 50 min, the solvent was removed in the evaporator. The solid product formed was crystallized from CH_2_Cl_2_ to give tetrabromide **13** (165 mg, 94%).

##### 2.3.2.1 2,5-Dibromo-1,6-bis(5-bromo-2-hydroxy-3,4-dimethoxyphenyl)hexane-1,6-dione (**13**)

Yellow solid, Mp 181–182 °C; *R*_f_ (10% EtOAc/hexane) 0.19; ^1^H NMR (400 MHz, CDCl_3_) δ (ppm): 12.08 (s, 2H, OH), 7.73 (s, 2H, aromatic), 5.13–5.05 (m, 2H, CHBr), 4.08 (s, 6H, OCH_3_), 3.92 (s, 6H, OCH_3_), 2.53–2.41 (m, 2H, CH_2_), 2.26–2.18 (m, 2H, CH_2_); ^13^C NMR (100 MHz, APT, CDCl_3_) δ (ppm): 196.6 (CO), 159.0 (C), 156.8 (C), 142.0 (C), 127.9 (CH), 114.4 (C), 106.6 (C), 61.6 (OCH_3_), 61.4 (OCH_3_), 45.1 (CHBr), 31.3 (CH_2_). IR (CH_2_Cl_2_, cm^–1^): 2917, 1626, 1428, 1377, 1306, 1268, 1186, 1129, 1055, 991, 958, 822, 801, 755, 736. HRMS (APCI-TOF) *m/z*: [M + H]^+^ calcd for C_22_H_22_^79^Br_4_O_8_: 730.8126; found: 730.8124.

## 3 Results and Discussion

### 3.1 Chemistry

**Scheme 1:**
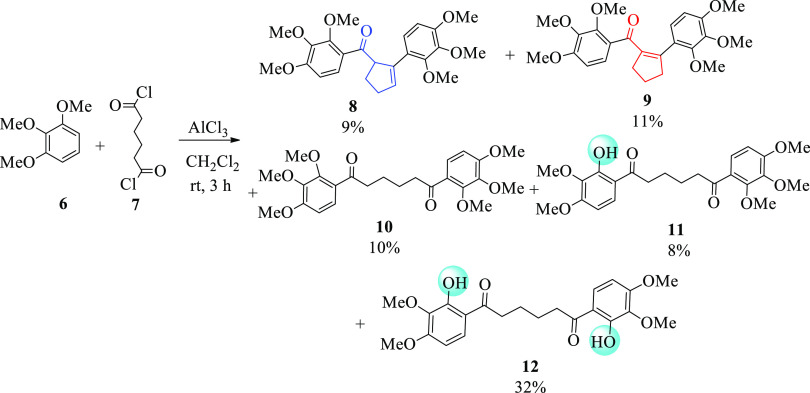
Reaction of 1,2,3-Trimethoxybenzene (**6**) with Adipoyl Chloride (**7**)

Initially, by the method described in our previous report,^15^ the reaction of 1,2,3-trimethoxybenzene (**6**) with adipoyl chloride (**7**) gave 1,6-bis(2,3,4-trimethoxyphenyl)hexane-1,6-dione (**10)**, its *ortho* demethylated derivatives **11** and **12**, and cyclocondensation derivatives **8** and **9** (Scheme 1). NMR spectra of purely isolated isomeric products are found in the Supporting Information. These products, which are also formed by the cyclocondensation reaction, contain different cyclopentene units. Although compounds **8** and **9** are isomeric structures, compound **9** has an α,β-unsaturated carbonyl structure while compound **8** does not. One of these products also contains double bond hydrogen (at 6.33 ppm), while the other does not. Of these isomeric products, it was determined that the one containing the double bond hydrogen was **8** and the other was **9**. Purification of such isomeric products by similar methods is also present in the literature.^15,16^

It is an expected product to form 1,4-dibenzoylbutane derivatives as a result of Friedel–Crafts acylation of a benzene derivative containing methoxy groups with adipoyl chloride in the presence of AlCl_3_. This reaction procedure is a standard organic chemistry procedure. Miyahara and Ito studied this acylation reaction of benzene with adipoyl chloride and observed the unconjugated enone **1** as well as 1,4-dibenzoylbutan. However, they did not observe the conjugated enone product.^10^ Kakemi et al. reported that bis(2-hydroxy-3,4-dimethoxyphenyl)alkane dione derivatives, which are the only demethylation products of methoxy groups in the ortho position, were formed by the Friedel–Crafts reaction with 1,2,3-trimethoxy benzene and dicarboxylic chloride derivatives having different chain lengths.^12^ They isolated only one demethylation product in the reaction of 1,2,3-trimethoxy benzene as well as various methoxy derivatives of benzene in the presence of adipoyl chloride and AlCl_3_.

However, in this study, in the reaction of 1,2,3-trimethoxy benzene with adipoyl chloride in the presence of AlCl_3_, five products were isolated, with 1,4-dibenzoylbutane (**10**) and two demethylation products **11** and **12**, as well as conjugated enone **9** and unconjugated enone **8** containing cyclopentene units. In terms of the organic synthesis procedure, this study is an exemplary study that can shed light on the formation of both demethylation and cyclization products, especially in the syntheses of 1,4-dibenzoylalkane containing methoxy groups in the ortho position.

**Scheme 2:**
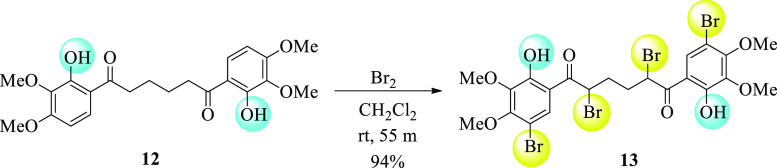
Reaction of Diketone **12** with Bromine

Cyclopentene derivatives **8** and **9** were observed in a cascade reaction, also known as a domino or tandem reaction. In the cascade reaction observed, first a Friedel–Crafts acylation reaction and then an aldol condensation reaction took place, which are widely used in organic synthesis. Although conjugated enone **9** is the main product in acid-catalyzed aldol condensation because it is thermodynamically more stable than unconjugated enone **8**, they were obtained in very close yields of 11 and 9%, respectively. One methoxy group in the ortho position was demethylated during the formation of **11**, while the methoxy groups in both ortho positions were demethylated during the formation of **12**. To investigate the effect of bromine on the intramolecular hydrogen bonds formed in these demethylation products, **13** was synthesized from the reaction of **12** with excess bromine (Scheme 2). All spectra and data for synthesized diketones **8**–**13** are compatible with those of their proposed structures.

### 3.2 Theoretical Calculations

#### 3.2.1 Molecular Structure and Stability

**Figure 2:**
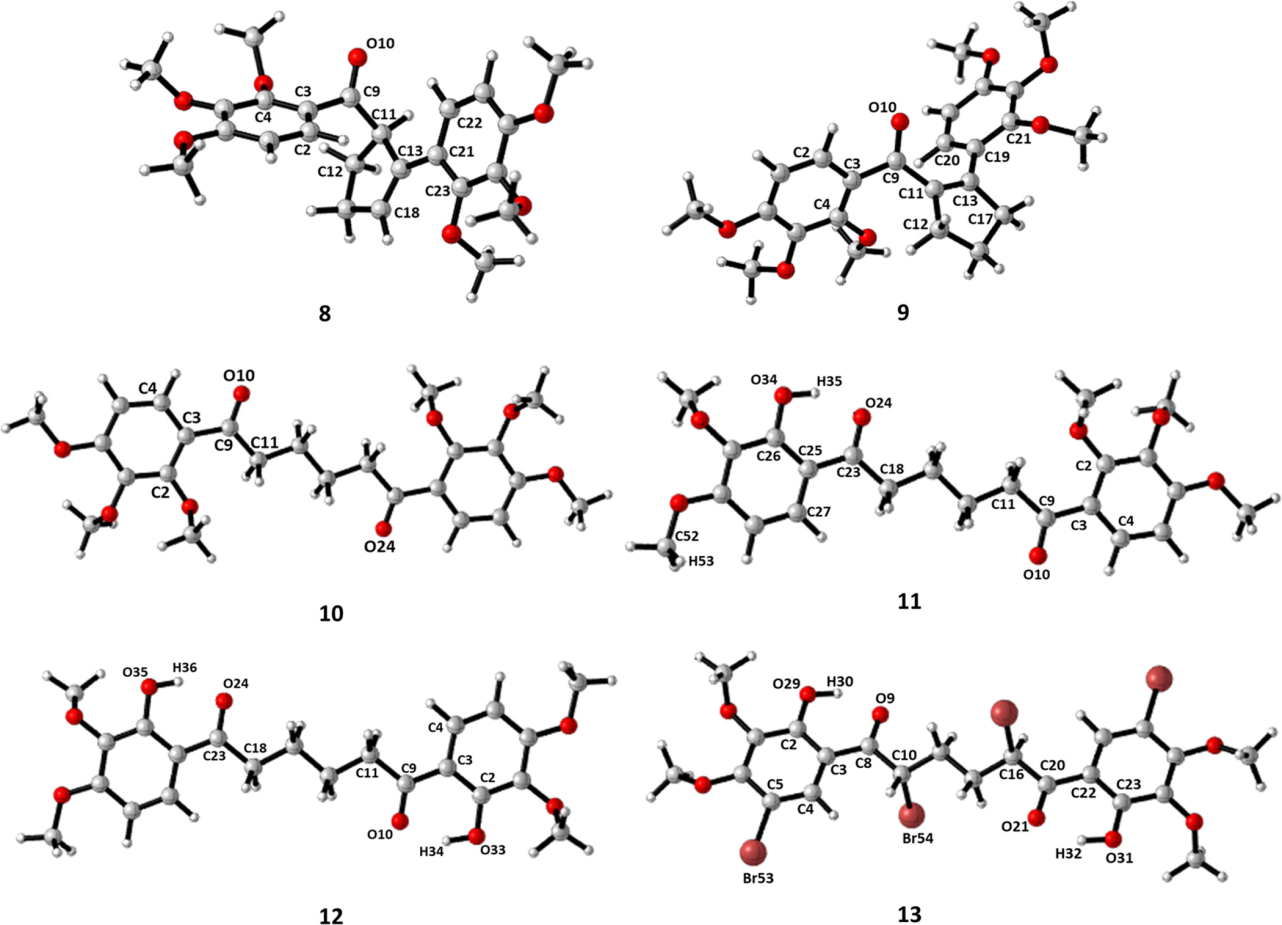
Optimized geometries of **8–13** using the B3LYP/6-311+G(d,p) basis set.

Cyclopentenes **8** and **9** and diketones **10–13** were optimized at the 6-311+G(d,p) level by two functional levels, B3LYP and M06-2X, of the DFT method in the gas phase. The optimized geometries of compounds **8**–**13** using the B3LYP/6-311+G(d,p) basis set are given in Figure 2. To compare the stabilities of conjugated enone **9** and unconjugated enone **8**, the structural parameters and total energies of their optimized structures were examined. The relative total energy for conjugated enone **9** at the B3LYP/6-311+G(d,p) level is 1.47 kcal/mol lower than that of unconjugated enone **8**. However, the relative energy value for **8** optimized at the M06-2X/6-311+G(d,p) level is 0.98 kcal/mol lower than that of **9**. In this case, the structural parameters of these compounds were investigated.

Some selected structural parameters of enones **8** and **9** and diketone **10–13** using the B3LYP/6-311+G(d,p) and M062X/6-311+G(d,p) basis set in the gas phase are given in the Supporting Information (Table S1). The bond length of double bond carbons C13–C18 calculated using B3LYP and M06-2X for **8** was 1.347 and 1.342 Å, respectively. The bond length of double bond carbons C11–C13 calculated using these two methods for **9** was 1.349 and 1.338 Å, respectively. The C18–C13–C21 bond angle formed between the double bond of the cyclopentene ring and the substituted phenyl group using B3LYP and M06-2X was 129.2° and 126.6° for **8**, respectively. The C11–C13–C19 bond angle formed between the double bond of the cyclopentene ring and the substituted phenyl group using B3LYP and M06-2X was 126.9° and 125.6° for **9**, respectively. For **9**, because the double bond of cyclopentene forms a conjugated system with the carbonyl group and the phenyl group, they must be in the same plane. The bonds that should be in the planar structure were examined using dihedral angles. Comparing the structural parameters calculated by the B3LYP method and the M06-2X method for compounds **8**–**13**, the bond angles and bond lengths were almost the same by the two methods.

The dihedral angles C2–C3–C9–O10 and C4–C3–C9–O10 using B3LYP and M06-2X for **8** and **9** are −114.9° and −113.0° and −32.0° and −15.1°, respectively. These values show that the carbonyl and phenyl groups for both compounds are not in the same plane because of weak conjugation. Similarly, the values of the dihedral angles C11–C13–C19–C21 and C11–C13–C19–C20 calculated by the B3LYP and M06-2X methods for **9** are 130.7° and 139.3° and −50.0° and −40.8°, respectively. According to these values, for **9**, the double bond in the cyclopentene ring and in the phenyl group attached to it are not in the same plane because of weak conjugation. The planes of the dihedral angles C2–C3–C9–O10 and C4–C3–C9–O10 according to the two methods for **10** are inclined by −173.5° and −165.8° and 7.4° and −15.4° from the skeleton of the molecular plane (Table S1). However, the dihedral angles formed by the carbonyl group and the phenyl group in compounds **11**–**13** are coplanar because their values are very close to 0° or 180°. While the dihedral angles C2–C3–C9–O10 and C4–C3–C9–O10 between the phenyl and carbonyl groups determined by the B3LYP and M06-2X methods for **12** are 0.2° and 0.6° and 179.6 and −179.5, respectively, these angles for **13** are −0.8° and −1.4° and 179.3° and 179.4°, respectively. In compound **13**, the bromine atom in the molecule weakened the conjugation and intramolecular hydrogen bond. The NBO analyses support these results.

The intramolecular hydrogen bond parameters for **11**–**13** are listed in Table 1. The structures of **11**–**13** are stabilized by intramolecular O–H···O hydrogen bond interactions formed between the hydrogen of the OH groups and the oxygen atoms of the carbonyl group (Table 1).

**Table 1:** Intramolecular Hydrogen Bonding Parameters of **11**–**13**

compounds	D–H···A	D–H (Å)		H···A (Å)		D**–**H···A (°)	
		B3LYP	M06-2X	B3LYP	M06-2X	B3LYP	M06-2X
**11**	O34–H35···O24	0.990	0.980	1.660	1.712	148.2	145.8
**12**	O33–H34···O10	0.990	0.979	1.666	1.721	148.0	145.6
**13**	O29–H30···O9	0.990	0.978	1.661	1.727	147.4	144.5

#### 3.2.2 NBO Analysis

NBO analysis, which is mostly used to investigate intramolecular interactions, hydrogen bonds, and charge transfers, is a useful theoretical method for measuring hyperconjugative interactions between atoms and molecules.^39,50,51^ A larger stabilization energy (E^2^) value shows a more intense interaction between electron donors and electron acceptors, leading to more conjugation in the system. The energies of these interactions can be calculated by second-order perturbation theory using the following equation:^50,51^
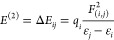
where *q_i_* is the donor orbital occupancy, ε_*j*_ and ε_*i*_ are diagonal elements, and *F*_(*i*,*j*)_ is the off-diagonal NBO Fock matrix element.

In the present study, we also investigated the energies of hyperconjugative interactions of intramolecular O–H···O hydrogen bondings by NBO analysis. Diketones **11–13** contain lone pairs of electrons on oxygen atoms of the carbonyl group, which results in intramolecular charge transfer, giving rise to stabilization of the molecular system.

Table 2 shows the donor–acceptor interaction energies for **11–13** calculated at the B3LYP/6-311+G(d,p) and M062X/6-311+G(d,p) level of theory. It can be clearly seen in Table 2 that the strongest stabilization energy for **11** occurred from the LP(2) O24 to antibonding orbitals σ* C18C23, σ* C23C25, and σ* O34H35 with an energy of 19.56, 12.86, and 20.81 kcal/mol at the B3LYP level, respectively. These energy values are 24.20, 16.53, and 17.02 kcal/mol at the M06-2X level. The strongest stabilization energy for **12** occurred from the LP(2) O24 to antibonding orbitals σ* O35H36 with an energy of 57.15 kcal/mol at the B3LYP level and 37.02 kcal/mol at the M06-2X level. The hyperconjugative interaction energy of LP(2) O10 → σ* C3C9, LP(2) O10 → σ* C9C11, LP(2) O10 → σ* O33H34, and LP(2) O24 → σ* C18C23 for **12** is 11.47, 18.20, 21.67, and 18.65 kcal/mol at the B3LYP level and 15.39, 22.01, 17.21, and 22.50 kcal/mol at the M06-2X level, respectively.

**Table 2:** NBO Donor–Acceptor Interactions for the Hydrogen Bondings and Intramolecular Interactions of **11–13** Using the B3LYP/6-311+G(d,p) and M06-2X/6-311+G(d,p) Basis Set

donor NBO (*i*)	acceptor NBO (*j*)	*E*^(2)^^a^ kcal/mol		*E*(*j*) – *E*(*i*)^b^ a.u.		*F*_(*i*,*j*)_^c^ a.u.	
		B3LYP	M06-2X	B3LYP	M06-2X	B3LYP	M06-2X
**11**							
LP(1) O24	BD*(1)C23C25	5.65	4.82	1.06	1.24	0.070	0.069
LP(1) O24	BD*(1)C25C27	2.32		0.53		0.031	
LP(1) O24	BD*(1)O34H35	3.58	3.60	1.13	1.32	0.057	0.062
LP(1) O24	BD*(1)C52H53	1.94	8.98	0.38	0.06	0.024	0.021
LP(1) O24	BD*(1)C25C26		1.75		0.13		0.015
LP(2) O24	BD*(1)C18C23	19.56	24.20	0.64	0.74	0.102	0.121
LP(2) O24	BD*(1)C23C25	12.86	16.53	0.70	0.85	0.086	0.107
LP(2) O24	BD*(1)C25C27	2.19		0.17		0.018	
LP(2) O24	BD*(1)O34H35	20.81	17.02	0.77	0.92	0.115	0.114
**12**							
LP(1) O10	BD*(1)C3C9	4.03	3.95	1.14	1.30	0.061	0.064
LP(1) O10	BD*(1)O33H34	3.42	3.39	1.08	1.27	0.055	0.059
LP(2) O10	BD*(1)C3C9	11.47	15.39	0.77	0.90	0.086	0.107
LP(2) O10	BD*(1)C9C11	18.20	22.01	0.70	0.84	0.103	0.123
LP(2) O10	BD*(1)O33H34	21.67	17.21	0.72	0.88	0.113	0.112
LP(1) O24	BD*(1)O35H36	5.93	5.15	0.64	0.80	0.055	0.058
LP(2) O24	BD*(1)C18C23	18.65	22.50	0.69	0.82	0.103	0.123
LP(2) O24	BD*(1)O35H36	57.15	37.02	0.28	0.41	0.114	0.111
**13**							
LP(1) O9	BD*(1)C3C8	4.05	3.90	1.15	1.31	0.061	0.064
LP(1) O9	BD*(1)O29H30		3.50		1.28		0.060
LP(2) O9	BD*(1)C3C8	11.49	15.61	0.78	0.90	0.086	0.108
LP(2) O9	BD*(1)C8C10	19.18	23.64	0.68	0.81	0.103	0.125
LP(2) O9	BD*(1)O29H30		15.98		0.88		0.108
LP(1) O21	BD*(1)C20C22	4.07	3.90	1.15	1.31	0.061	0.064
LP(1) O21	BD*(1)O31H32		3.50		1.28		0.060
LP(2) O21	BD*(1)C16C20	19.20	23.64	0.68	0.81	0.104	0.125
LP(2) O21	BD*(1)C20C22	11.47	15.61	0.78	0.90	0.086	0.108
LP(2) O21	BD*(1)O31H32		15.97		0.87		0.108

a *E*^(2)^ means energy of hyperconjugative interactions (stabilization energy).

b Energy difference between the donor and acceptor *i* and *j* NBO orbitals.

c *F*_(*i*,*j*)_ is the Fock matrix element between *i* and *j* NBO orbitals.

The strongest stabilization energy for **13** occurred from the LP(2) O21 to antibonding orbitals σ* C16C20 with an energy of 19.20 kcal/mol at the B3LYP level and 23.6 kcal/mol at the M06-2X level. The stabilization energy of LP(2) O9 → σ* C3C8, LP(2) O9 → σ* C8C10, and LP(2) O21 → σ* C20C22 for **13** is 11.49, 19.18, and 11.47 kcal/mol at the B3LYP level and 15.61, 23.64, and 15.61 kcal/mol at the M06-2X level, respectively.

In Table 2, it can be clearly seen that the bromine atom in the compound **13** weakens the hydrogen bond by lowering the stabilization energy formed between the oxygen atom of the carbonyl group and the hydrogen atom of the OH group. All charges on atoms by NBO analysis using B3LYP/6-311+G(d,p) and M062X/6-311+G(d,p) basis sets for compounds **8**–**13** are given in the Supporting Information (Table S3).

#### 3.2.3 NMR Spectral Analysis

**Figure 3:**
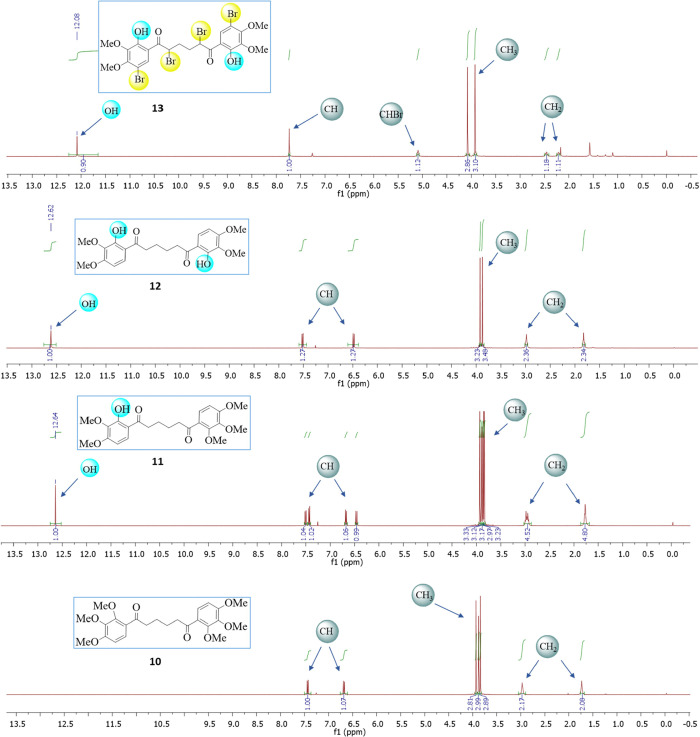
^1^H NMR spectra of **10–13**.

**Figure 4:**
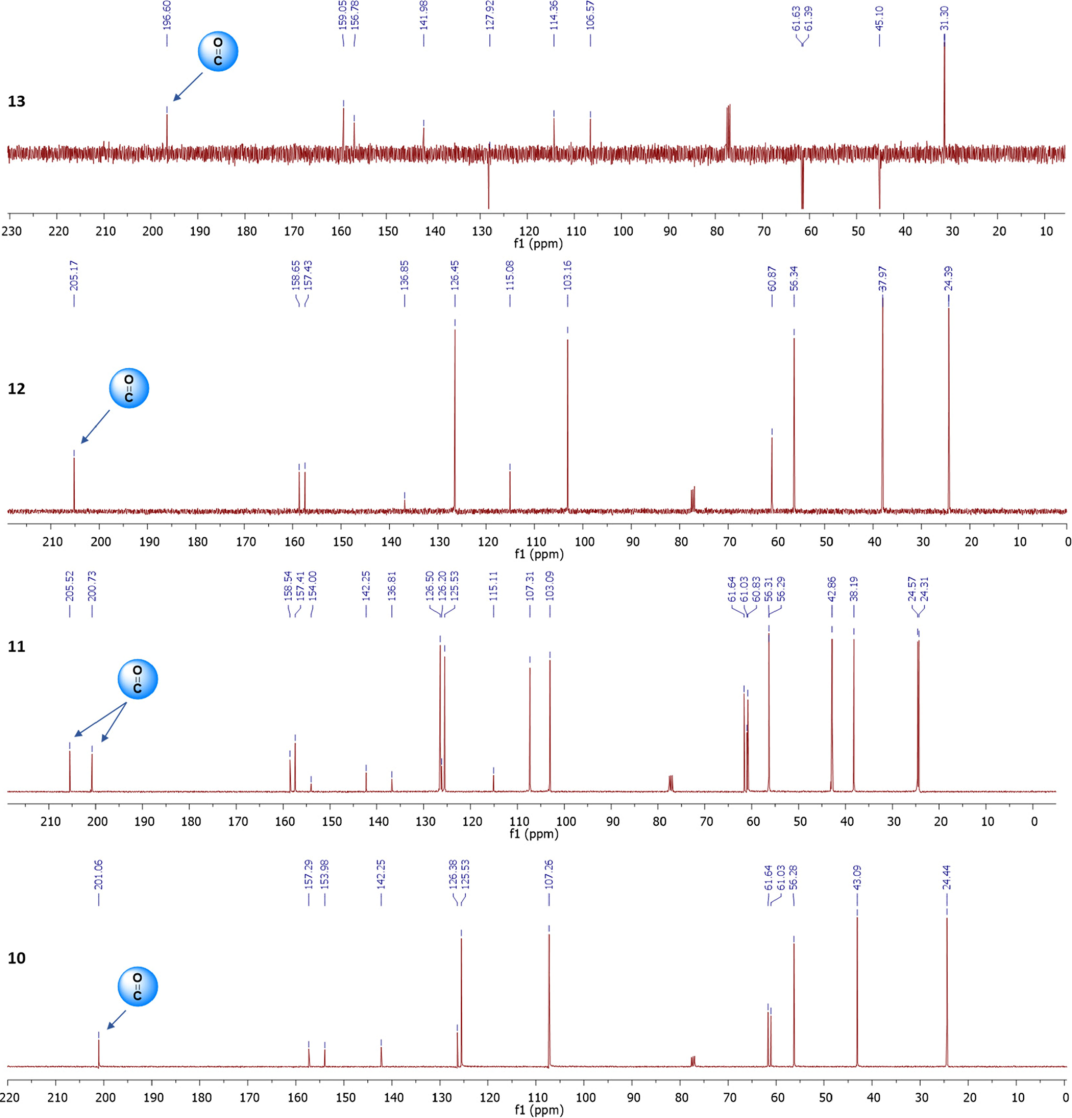
^13^C NMR spectra of **10–13**.

The ^1^H and ^13^C chemical shift calculations of **10**–**13** were performed using the GIAO method^48,49^ by B3LYP and M06-2X with the 6-311+G(d,p) basis set in the CDCl_3_ with respect to tetramethylsilane (TMS). Experimental ^1^H and ^13^C NMR spectra of **10**–**13** are given in Figures 3 and 4, respectively. In phenolic compounds containing a carbonyl group in the ortho position, strong intramolecular hydrogen bonds are formed between the oxygen atom of the carbonyl group and the hydrogen atom of the OH group. This OH proton forming the strong hydrogen bond resonates as a singlet in the ^1^H NMR spectrum in the range of about 12–13 ppm.^21^ When this hydrogen bond is stronger, it resonates in the downfield (toward 13 ppm), and when it is weaker, it resonates in the upfield (toward 12 ppm). On the other hand, phenolic OH protons generally resonate between at 6 and 10 ppm.^52^ In compounds **11** and **12**, the OH protons resonate in the lower field, while this proton in compound **13** resonates in the higher field. This shows that the hydrogen bond in compound **13** is weaker than in compounds **12** and **13**. Surprisingly, stronger hydrogen bonds were expected because of the inductive effect of the bromine atoms at the alpha position of the carbonyl group in compound **12**, while weaker hydrogen bonds were formed. Similarly, in the ^13^C NMR spectra of compounds **11** and **12**, the carbon atom of the carbonyl group forming the hydrogen bond resonated in the lower field, while this carbon atom in the compound **13** resonated in the higher field. Computational studies have also been carried out to support these experimental data. Because the bromine atom in the molecule weakens the hydrogen bond, the calculated chemical shift values of the carbon atom in the carbonyl group and the hydrogen atoms in the hydroxyl group were compared with the experimental values (Table 3). Both atoms were observed in the most downfield region in the experimental and calculated ^1^H and ^13^C NMR spectra. As seen in Table 3, the experimental resonance signals of the −OH protons for **11** (H35), **12** (H34, H36), and **13** (H30, H32) were the most downfield signals at δ 12.64, 12.62, and 12.08 ppm as a singlet in the ^1^H NMR spectra, while the theoretical chemical shift values for these hydrogen atoms were obtained at δ 12.95, 12.89, and 12.77 ppm at the B3LYP level and δ 12.84, 12.60, and 12.04 ppm at the M062X level. In the experimental ^13^C NMR spectra, chemical shift values for **10** (C9 or C23), **11** (C9, C23), **12** (C9 or C23), and **13** (C8 or C20) were observed in the most downfield signal at 201.06, 200.73–205.52, 205.17, and 196.60, respectively. The theoretical chemical shift values for these carbon atoms of carbonyl groups were obtained at δ 207.09, 206.56, 214.53, 213.92, and 206.45 ppm at the B3LYP level and δ 220.98, 219.00, 229.07, 228.00, and 219.53 ppm at the M062X level, respectively.

**Table 3:** Experimental and Calculated ^1^H and ^13^C Isotropic Chemical Shift Values (with Respect to TMS, All Values in ppm) of Hydroxyl Hydrogen and Carbonyl Carbon of **10–13**

compound	atom	B3LYP	M06-2X	experimental
**10**	C9, C23	207.09	220.98	201.06
**11**	C9	206.56	219.00	200.73
	C23	214.53	229.07	205.52
	H35	12.95	12.84	12.64
**12**	C9, C23	213.92	228.00	205.17
	H34, H36	12.89	12.60	12.62
**13**	C8, C20	206.45	219.53	196.60
	H30, H32	12.77	12.04	12.08

The maximum deviation from the experimental chemical shift for the ^13^C NMR chemical shift is 9.85 ppm for **13** (C8, C20) at the B3LYP level and 23.55 ppm for **11** (C23) at the M062X level. In the ^1^H NMR chemical shift, the maximum deviation from the experimental value is 0.69 ppm for **13** (H30, H32) at the B3LYP level and 0.2 ppm for **11** (H35) at the M062X level. In general, when compared with the experimental data, it was observed that the B3LYP level gave closer ^13^C NMR values, while the M062X level gave more consistent ^1^H NMR values. The calculated ^1^H NMR and ^13^C NMR chemical shift values of **8**–**13** (Table S2) and the experimental NMR spectra of all compounds are presented in the Supporting Information.

#### 3.2.4 FMO Analysis

**Figure 5:**
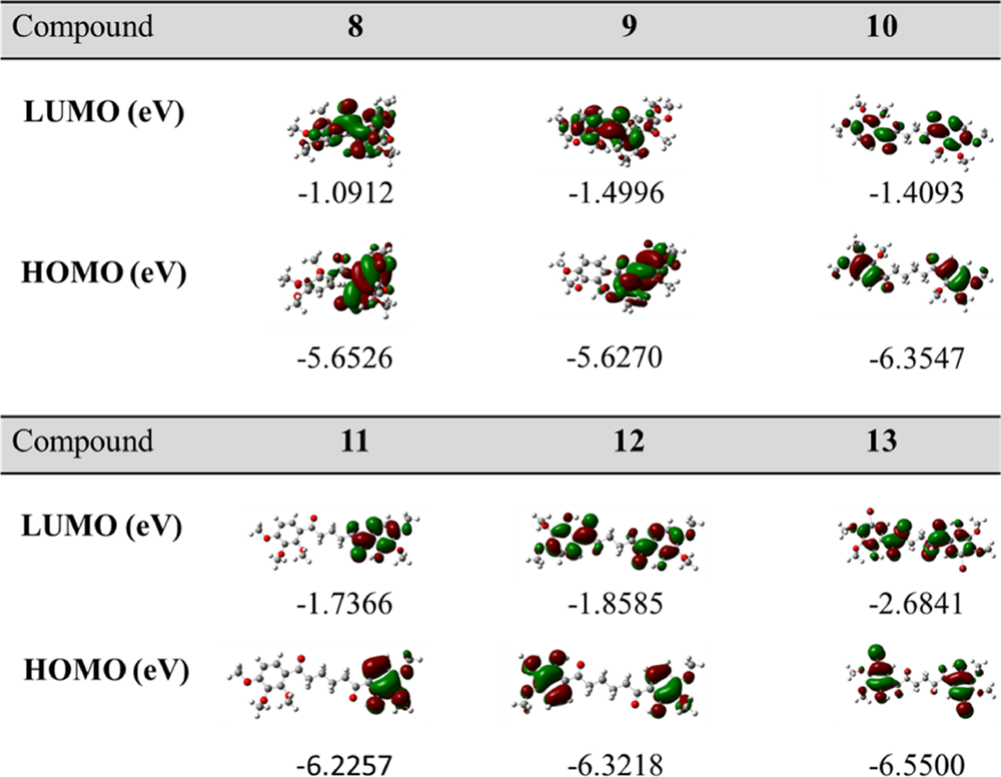
Frontier molecular orbitals of **8–13** using the B3LYP/6-311+G(d,p) basis set.

FMO analysis is widely used to predict the kinetic stability of molecules and the most reactive sites in conjugated systems. The stability and reactivity of a molecule are related to the difference in the highest occupied molecular orbital (HOMO)–lowest unoccupied molecular orbital (LUMO) energy gap (Δ*E* = |*E*_HOMO_ – *E*_LUMO_|). FMO analysis was performed for **8**–**13** using the B3LYP/6-311+G(d,p) and M062X/6-311+G(d,p) basis set in the gas phase (Figure 5). The HOMO and LUMO energy values for **8**–**13** were calculated in eV (Figure 5). The frontier energy gaps (Δ*E*) of **8–13** are 4.5614, 4.1274, 4.9454, 4.4891, 4.4633, and 3.8659 eV, respectively (Table 4). There is an important relationship between chemical stability and the energy gap between the HOMO and the LUMO. Chemical stability generally tends to increase with the large values of the energy gap (Δ*E* = *E*_LUMO_ – *E*_HOMO_). Accordingly, we can presume that compound **10** (Δ*E* = 4.9454 eV) is the most stable compound, while compound **13** is the most reactive compound (Δ*E* = 3.8659 eV) (Table 4).

**Table 4:** Quantum Chemical Reactivity Identifiers (eV) of **8–13**

parameters	**8**	**9**	**10**	**11**	**12**	**13**
*I*	5.6526	5.6270	6.3547	6.2257	6.3218	6.5500
*A*	1.0912	1.4996	1.4093	1.7366	1.8585	2.6841
Δ*E*	4.5614	4.1274	4.9454	4.4891	4.4633	3.8659
η	2.2807	2.0637	2.4727	2.2446	2.2317	1.9330
σ	0.4385	0.4846	0.4044	0.4455	0.4481	0.5173
χ	3.3719	3.5633	3.8820	3.9812	4.0902	4.6170
μ	–3.3719	–3.5633	–3.8820	–3.9812	–4.0902	–4.6170
ω	2.4926	3.0763	3.0473	3.5307	3.7482	5.5139

In the case of the HOMO, the charge density for **8** and **9** is mainly accumulated on the cyclopentene ring and its substituted phenyl ring. There is a very small contribution on the carbonyl group. However, in the case of the LUMO, the charge density for **8** spreads out from the phenyl group toward the other phenyl group, while the charge density for **9** spreads out from the cyclopentene ring toward the benzoyl group. In the case of the LUMO, the charge density for **11** spreads out on the benzoyl group with the phenol ring. In the HOMO, the density is only on the phenol ring. In symmetric compounds **10**, **12**, and **13**, these charge densities spread out from the aromatic ring to the carbonyl group. The energy gap in compound **13**, which is formed by adding four bromine atoms to compound **12**, decreased by 0.5 eV. In this case, we can consider that the reactivity of compound **13** is greater than that of compound **12**.

Global reactivity descriptors are important quantum chemical parameters used to predict the stability or reactivity of molecules. According to Koopman’s theorem,^53^ ionization energy (*I* = −HOMO) and electron affinity (*A* = −LUMO) are related to the HOMO and LUMO energies. In this paper, we also present values of reactivity descriptors such as global hardness (η), global softness (σ), electronegativity (χ), chemical potential (μ), and electrophilicity (ω) calculated by the following equations (Table 4):^54−56^
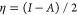

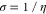

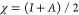

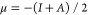

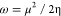


#### 3.2.5 Molecular Electrostatic Potential

**Figure 6:**
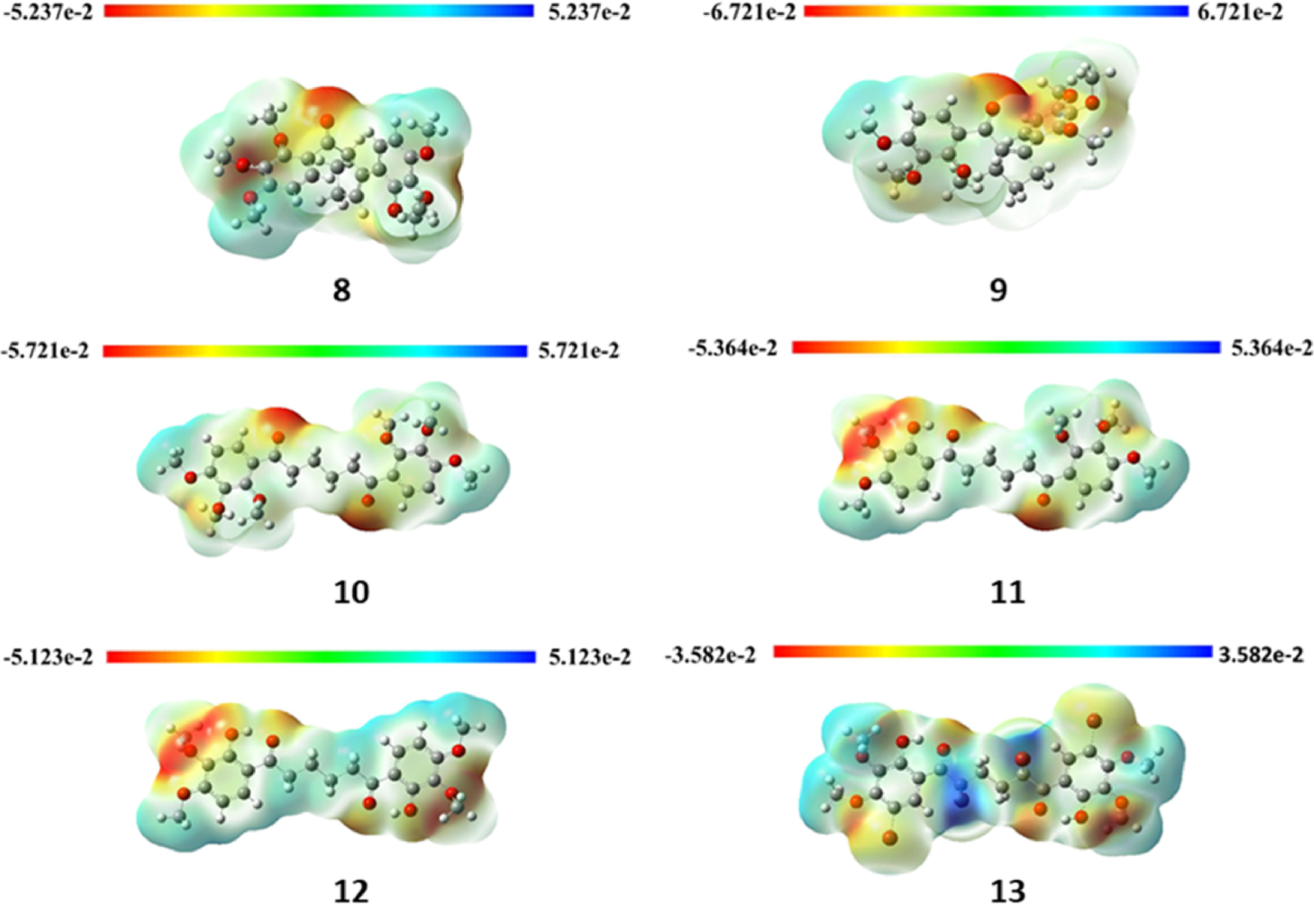
MEP surfaces of **8–13** using the B3LYP/6-311+G(d,p) basis set.

The MEP surfaces of **8–13** are illustrated in Figure 6. The MEP gives visual information about determining reactive sites for electrophilic and nucleophilic attacks and hydrogen bond interactions as well as total charge density formed in the molecule.^57^ Red depicts the electron-poor regions (negative side) in the molecule, while blue shows the electron-rich areas (positive side).

The electron-poor red regions in compounds **8**–**10** are mainly located around the carbonyl group. On the other hand, the electron-rich regions (blue) are on the benzene rings (for **8** and **10**), cyclopentene (for **8** and **9**), and CH_2_ groups (for **10**) (Figure 6). In compounds **11** and **12**, the electron-poor regions are located on the carbonyl groups and the methoxy groups adjacent to the hydroxyl groups, while in compound **13**, the regions where the carbon atoms to which the bromine is attached are quite rich in electrons.

## 4 Conclusions

We synthesized two isomeric cyclopentene derivatives (**8** and **9**) and three 1,4-dibenzoylbutane derivatives (**10**–**12**) starting from 1,2,3-trimethoxybenzene with adipoyl chloride in the presence of AlCl_3_ in just one step. The formation of compounds **8** and **9** occurs by a cascade reaction, also known as a domino reaction or tandem reaction, resulting from the Friedel–Crafts reaction and then aldol condensation. The OH groups in the diketones **11** and **12** were formed by regioselective demethylation of the methoxy groups because they are at the ortho position of the carbonyl groups. These OH groups also form strong hydrogen bonds. Tetrabromide **13** was synthesized from the reaction of diketone **12** with excess bromine. All compounds were optimized by means of DFT calculations using two functional levels, B3LYP and M06-2X, with the 6-311+G(d,p) basis set. The same method was used to calculate the structural parameters and for NBO, FMO, and MEP analysis of all compounds, as well as for the GIAO approach for calculating the NMR chemical shifts. It was observed that the bromine atom in tetrabromide **13** weakened the conjugation and intramolecular hydrogen bonding in the molecule.

In terms of the organic synthesis procedure, this study is an exemplary study that can shed light on the formation of both demethylation and cyclization products, especially in the syntheses of 1,4-dibenzoylalkane containing methoxy groups in the ortho position.

## Supporting Information Available

Spectroscopic data (^1^H and ^13^C NMR spectra) of the products; selected structural parameters, cartesian coordinates; and NBO charges for the optimized structures (PDF)

## Notes

All DFT calculations were performed with the software package Gaussian 09W. The results were visualized by the GaussView 5 and CYLview software for preparation. The details of these methods and software are provided in the Calculation details section, including relevant citations. All calculations including the selected structural parameters, ^1^H and ^13^C NMR chemical shifts, NBO charges, and cartesian coordinates of all optimized structures are given in the Supporting Information.
