# Supplementary material for: AlCl3-Catalyzed Cascade Reactions of 1,2,3-Trimethoxybenzene and Adipoyl Chloride: Spectroscopic Investigations and Density Functional Theory Studies
Source: ACS Omega. 2022 Oct 14;7(43):38882–93. doi: 10.1021/acsomega.2c04612 (PMC9631882; doi:10.1021/acsomega.2c04612)
Supplement: Supplementary file 1 — ao2c04612_si_001.pdf [file ao2c04612_si_001.pdf]

Supporting Information

$AlCl_3$-catalyzed cascade reactions of 1,2,3-trimethoxybenzene and adipoyl chloride: Spectroscopic investigations and Density Functional Theory Studies

*Yasin Çetinkaya**, *Tekin Artunç, Abdullah Menzek**

Department of Chemistry, Faculty of Science, Atatürk University, 25240 Erzurum, Turkey

Corresponding Authors

Yasin Çetinkaya - orcid.org/0000-0001-6617-5055

Phone: +904422314389; Email: yasin.cetinkaya@atauni.edu.tr

Abdullah Menzek - orcid.org/0000-0001-6177-7532

Phone: +904422314423; Email: amenzek@atauni.edu.tr

Contents **Page**

Table S1. Selected structural parameters of **8-13** S2

Figure S1-S6. ^{1}H NMR and ^{13}C NMR spectra of **8-13** S4

Table S2. ^{1}H and ^{13}C chemical shift values of **8-13** calculated with the GIAO method S10

Table S3. Charges accumulated on all atoms with NBO analysis of **8-13** S23

Table S4. Cartesian coordinates for the optimized structures of **8-13** S38

Table S1. Selected structural parameters of **8-13**.

	B3LYP	M062X		B3LYP	M062X
**8**			**9**		
Bond lengths (Å)					
C3-C9	1.508	1.505	C3-C9	1.507	1.504
C9-O10	1.216	1.208	C9-O10	1.222	1.212
C9-C11	1.535	1.525	C9-C11	1.490	1.491
C13-C21	1.475	1.474	C13-C19	1.478	1.474
C13-C18	1.347	1.342	C11-C13	1.349	1.338
Bond angles (°)					
C2-C3-C9	121.9	122.6	C2-C3-C9	117.0	117.3
C4-C3-C9	118.9	117.9	C4-C3-C9	124.4	124.2
C3-C9-O10	119.8	120.5	C3-C9-O10	118.6	119.5
O10-C9-C11	120.4	121.6	O10-C9-C11	121.4	121.0
C11-C13-C18	110.2	110.4	C9-C11-C13	126.1	126.4
C18-C13-C21	129.2	129.6	C9-C11-C12	121.8	121.3
C13-C21-C22	119.9	120.0	C11-C13-C19	126.9	125.6
Dihedral angles (°)					
C2-C3-C9-O10	-114.9	-113.0	C2-C3-C9-O10	-32.0	-15.1
C4-C3-C9-O10	60.9	65.0	C4-C3-C9-O10	145.3	161.4
C18-C13-C21C22	-171.2	-169.2	O10-C9-C11-C13	-38.4	-61.0
C18-C13-C21-C23	9.189	10.407	C11-C13-C19-C21	130.7	139.3
			C11-C13-C19-C20	-50.0	-40.8
	B3LYP	M062X		B3LYP	M062X
**10**			**11**		
Bond lengths (Å)					
C3-C9	1.508	1.504	C3-C9	1.507	1.504
C9-O10	1.220	1.212	C9-O10	1.220	1.212
C9-C11	1.520	1.516	C23-C124	1.238	1.491
			C23-C25	1.468	1.474
			O34-H35	0,990	0.980
Bond angles (°)					
C2-C3-C9	126.4	125.1	C2-C3-C9	126.4	125.5
C4-C3-C9	116.1	116.8	C4-C3-C9	116.1	116.7
C3-C9-O10	118.8	119.1	C3-C9-O10	119.0	119.1
O10-C9-C11	120.0	120.4	C26-O34-H35	106.4	107.2
			C26-C25-C23	119.6	119.7
			C25-C23-024	120.7	120.9
Dihedral angles (°)					
C2-C3-C9-O10	-173.5	-165.8	C2-C3-C9-O10	179.4	178.3
C4-C3-C9-O10	7.4	-15.4	C4-C3-C9-O10	0.298	-0.8
			O34-C26-C25-C23	-0.1	0.3
			C26-C25-C23-O24	-0.1	-0.4
			C27-C25-C23-O24	-179.4	-180.0
	B3LYP	M062X		B3LYP	M062X
**12**			**13**		
Bond lengths (Å)					
C3-C9	1.467	1.473	C3-C8	1.462	1.468

C9-O10	1.238	1.226	C8-O9	1.236	1.223
O33-H34	0.990	0.979	O29-H30	0.990	0.978
			C5-Br53	1.911	1.890
			C10-Br54	2.010	1.972
Bond angles (º)					
C2-C3-C9	119.6	119.7	C2-C3-C8	118.9	119.1
C4-C3-C9	122.4	121.9	C4-C3-C8	122.9	122.3
C3-C9-O10	120.9	121.1	C3-C8-O9	121.5	121.9
O10-C9-C11	119.2	119.5	O9-C8-C10	117.8	118.0
			C4-C5-Br53	119.6	120.0
Dihedral angles (º)					
C2-C3-C9-O10	0.2	0.6	C2-C3-C8-O9	-0.8	-1.4
C4-C3-C9-O10	179.6	-179.5	C4-C3-C8-O9	179.3	179.4
C4-C3-C2-O33	-179.2	179.8	C4-C3-C2-O29	-179.7	179.1
			C3-C4-C5-Br53	179.0	179.6

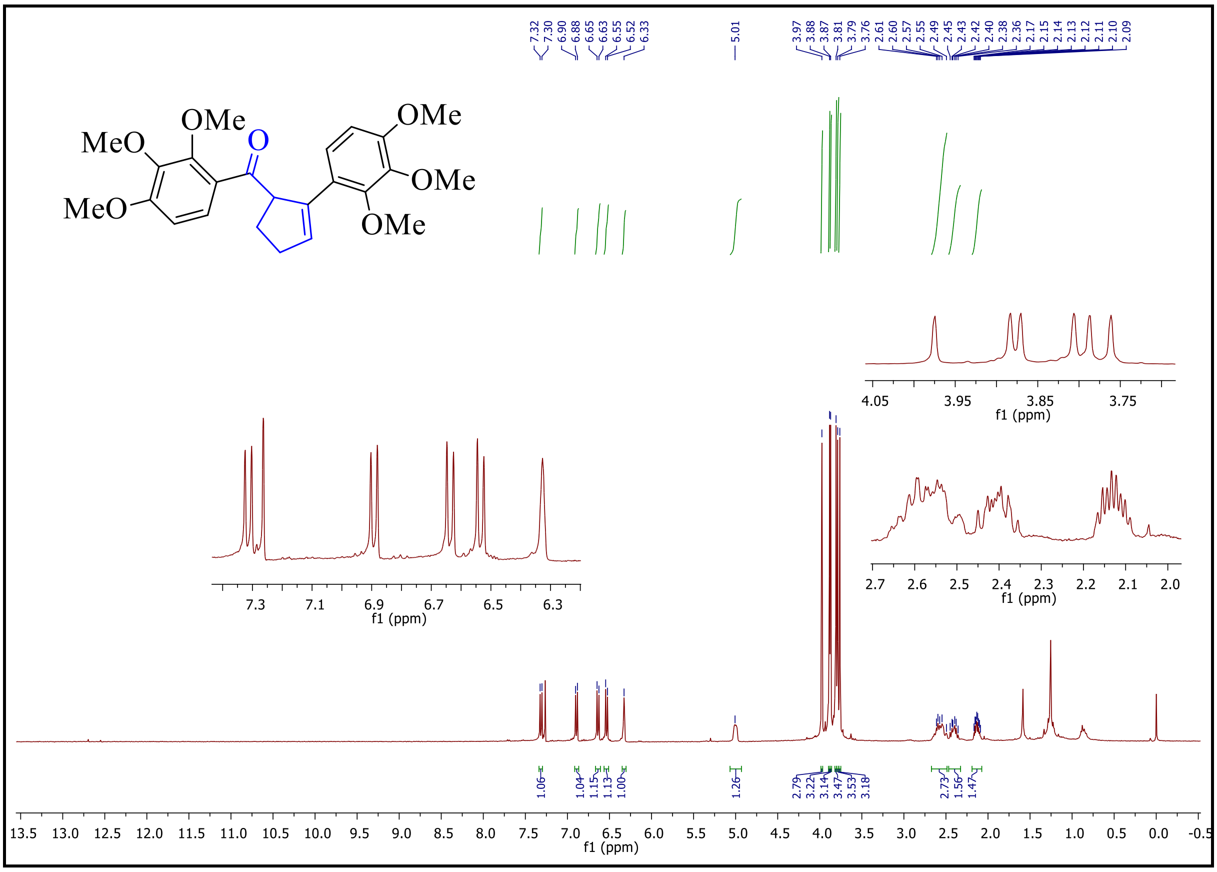

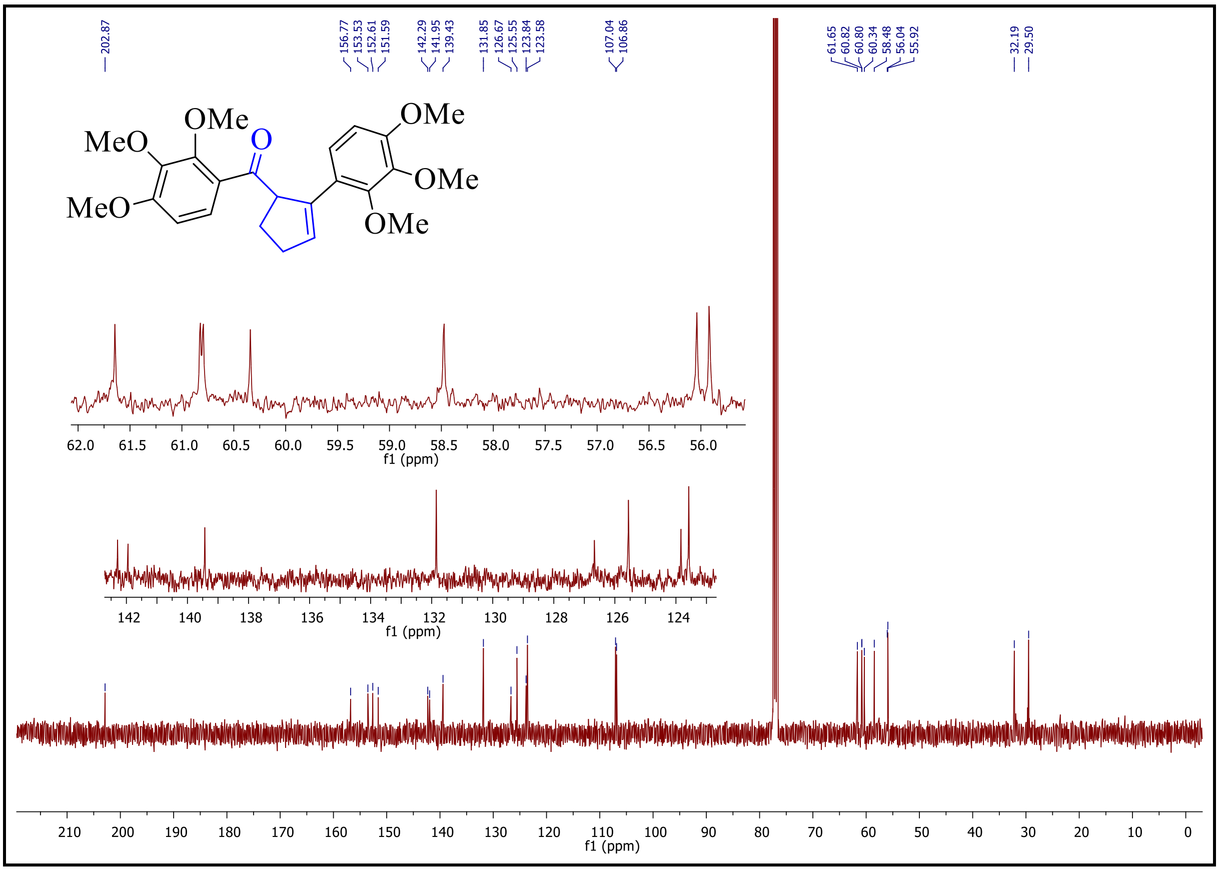

Figure S1. ^{1}H NMR and ^{13}C NMR spectra of **8**.

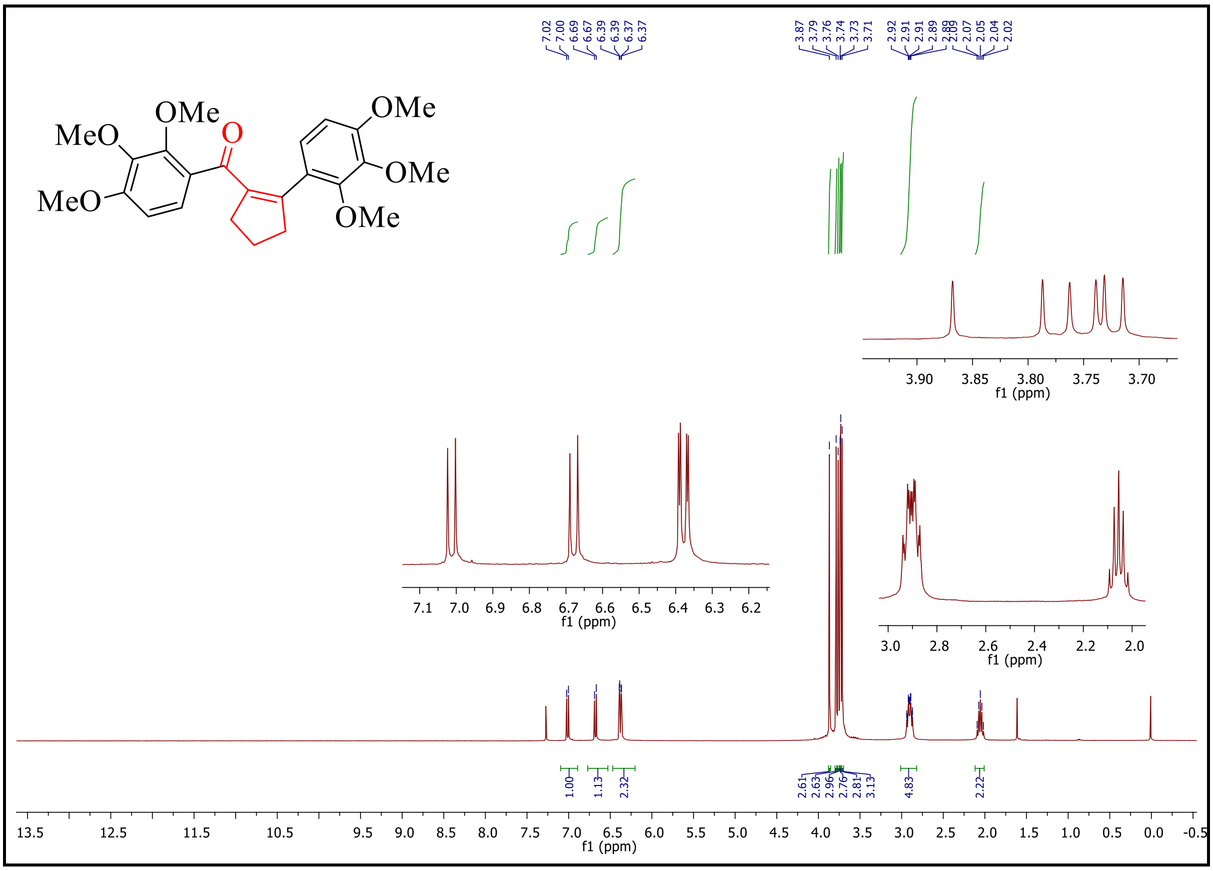

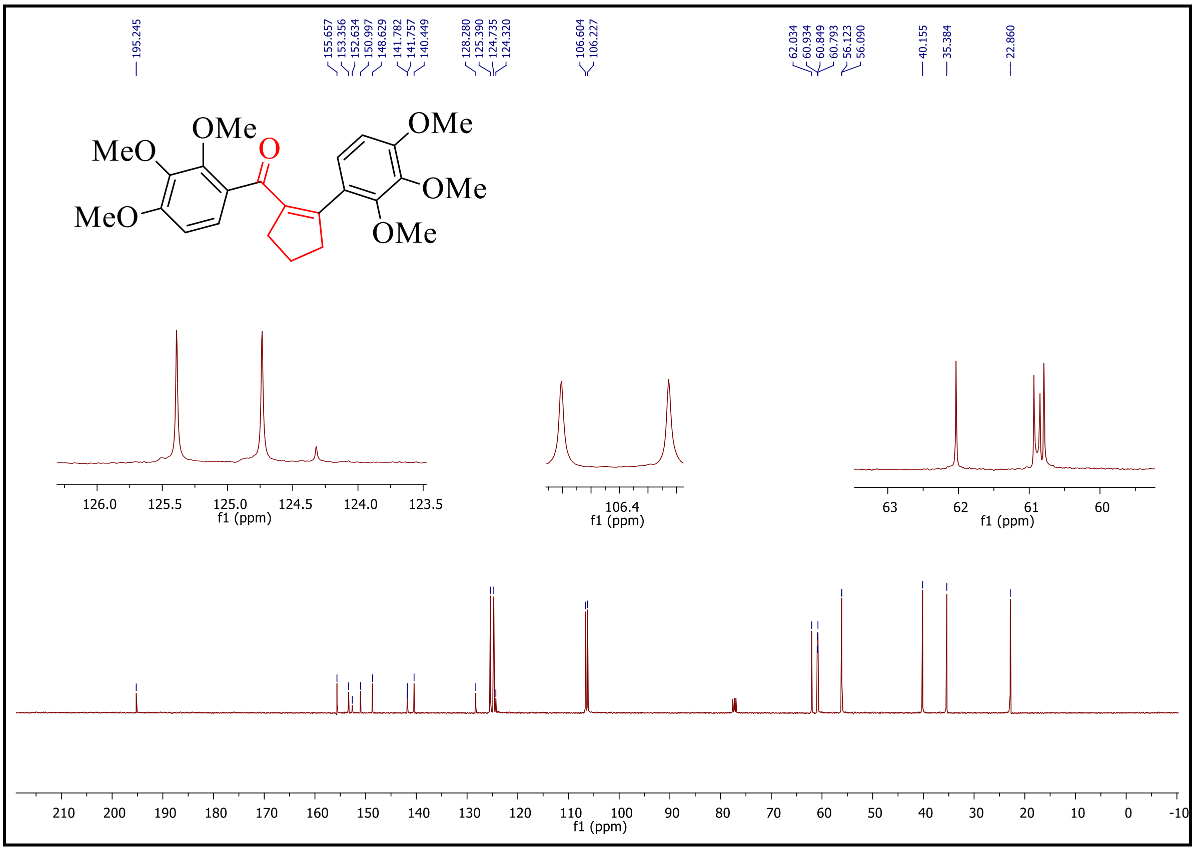

Figure S2. ^{1}H NMR and ^{13}C NMR spectra of **9**.

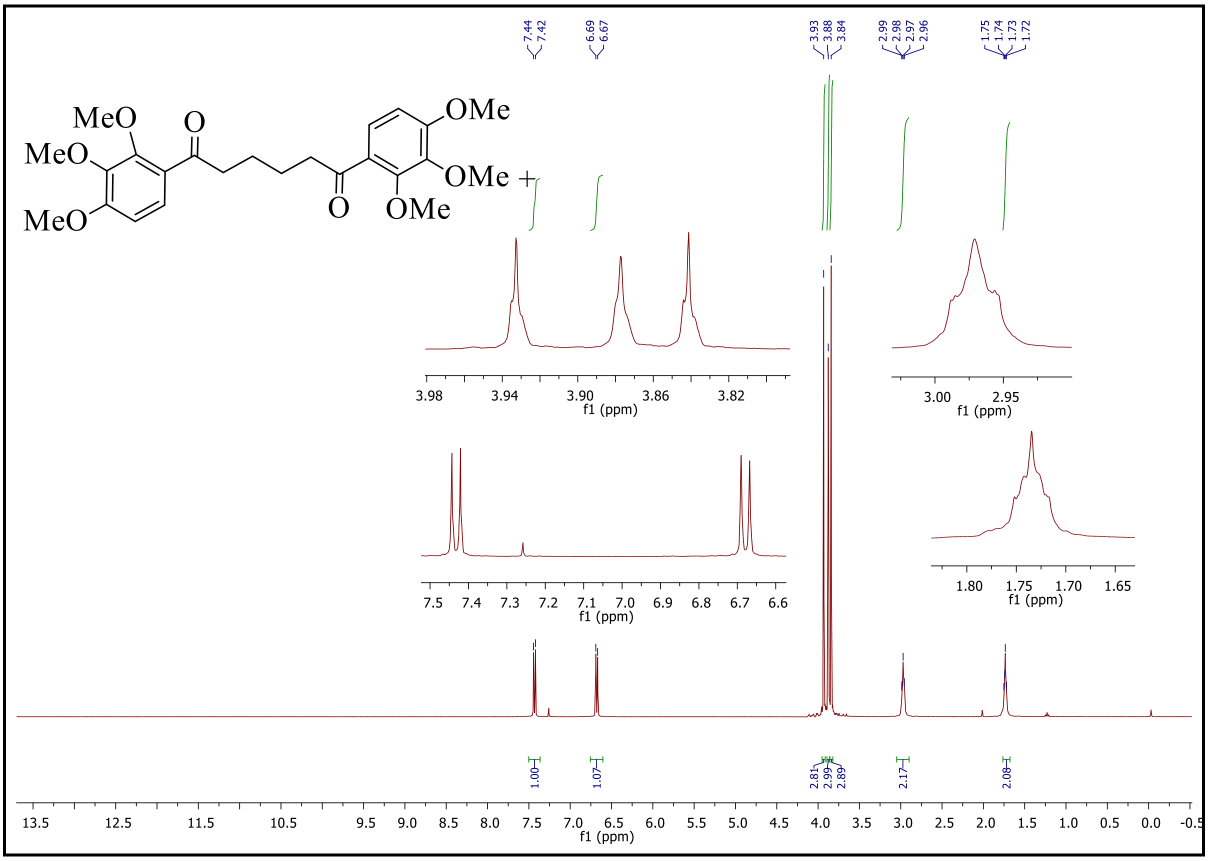

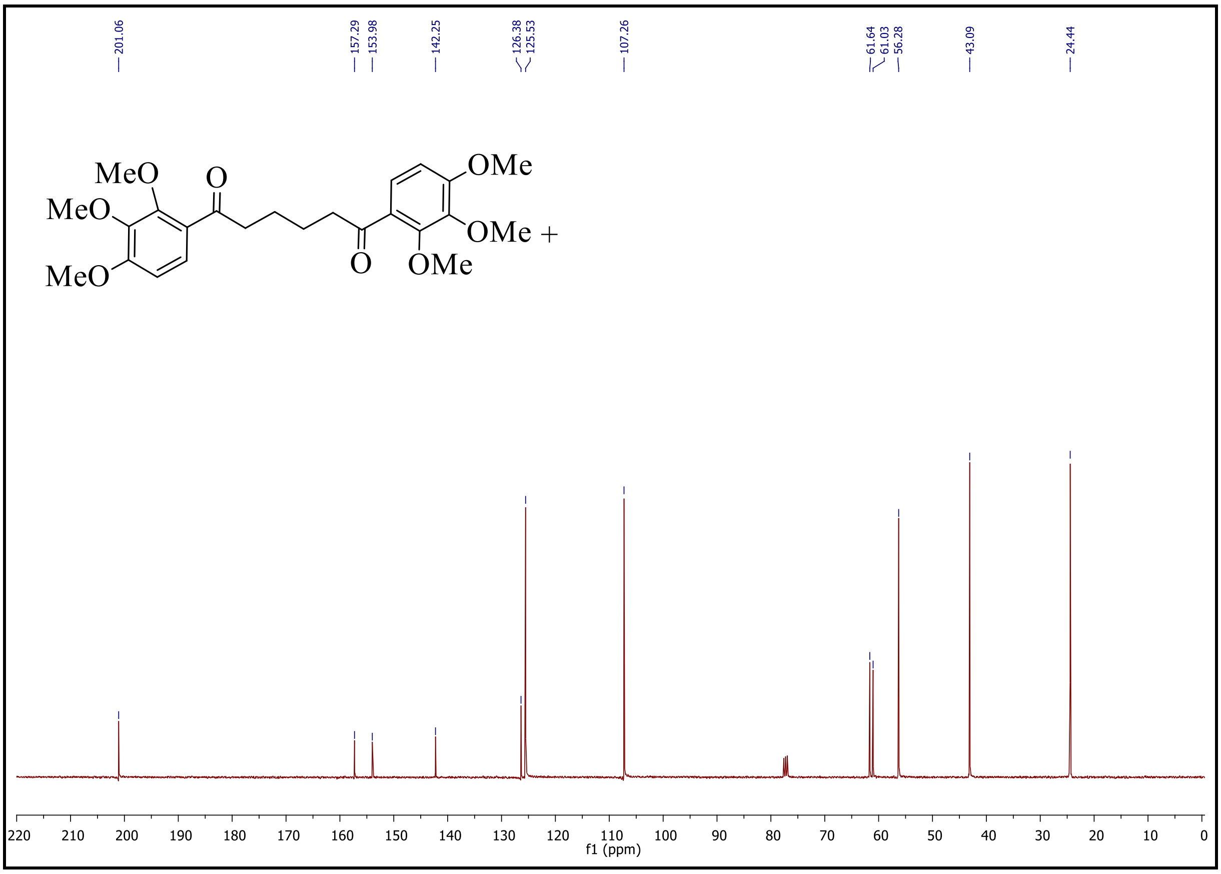

Figure S3. ^{1}H NMR and ^{13}C NMR spectra of **10**.

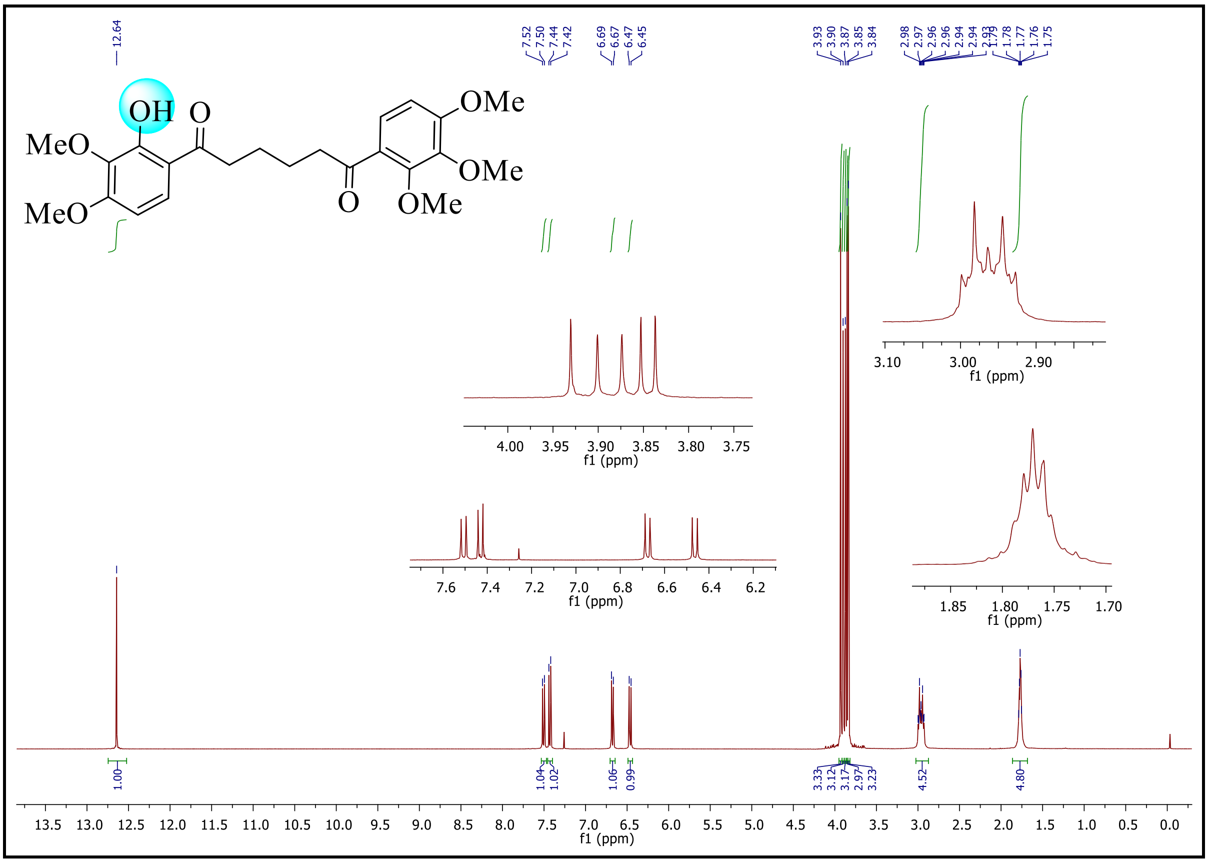

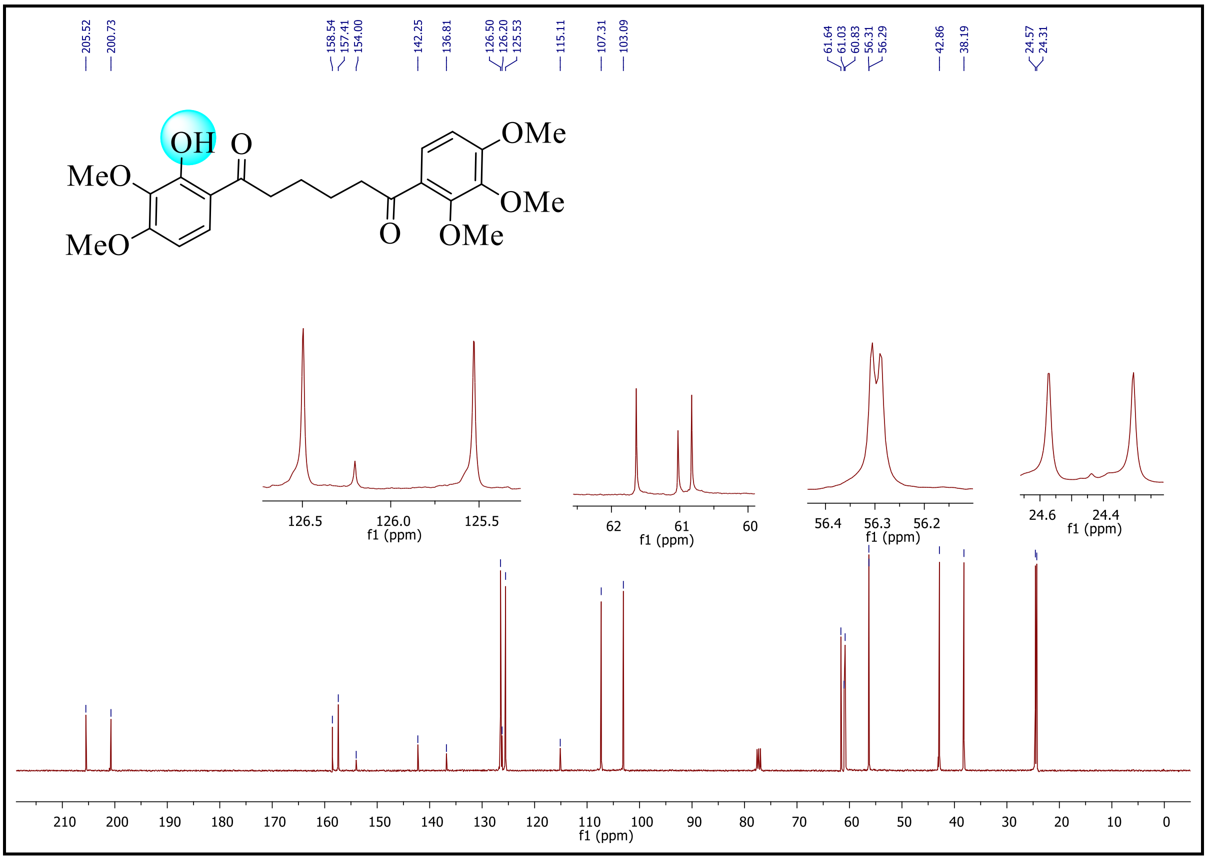

Figure S4. ^{1}H NMR and ^{13}C NMR spectra of **11**.

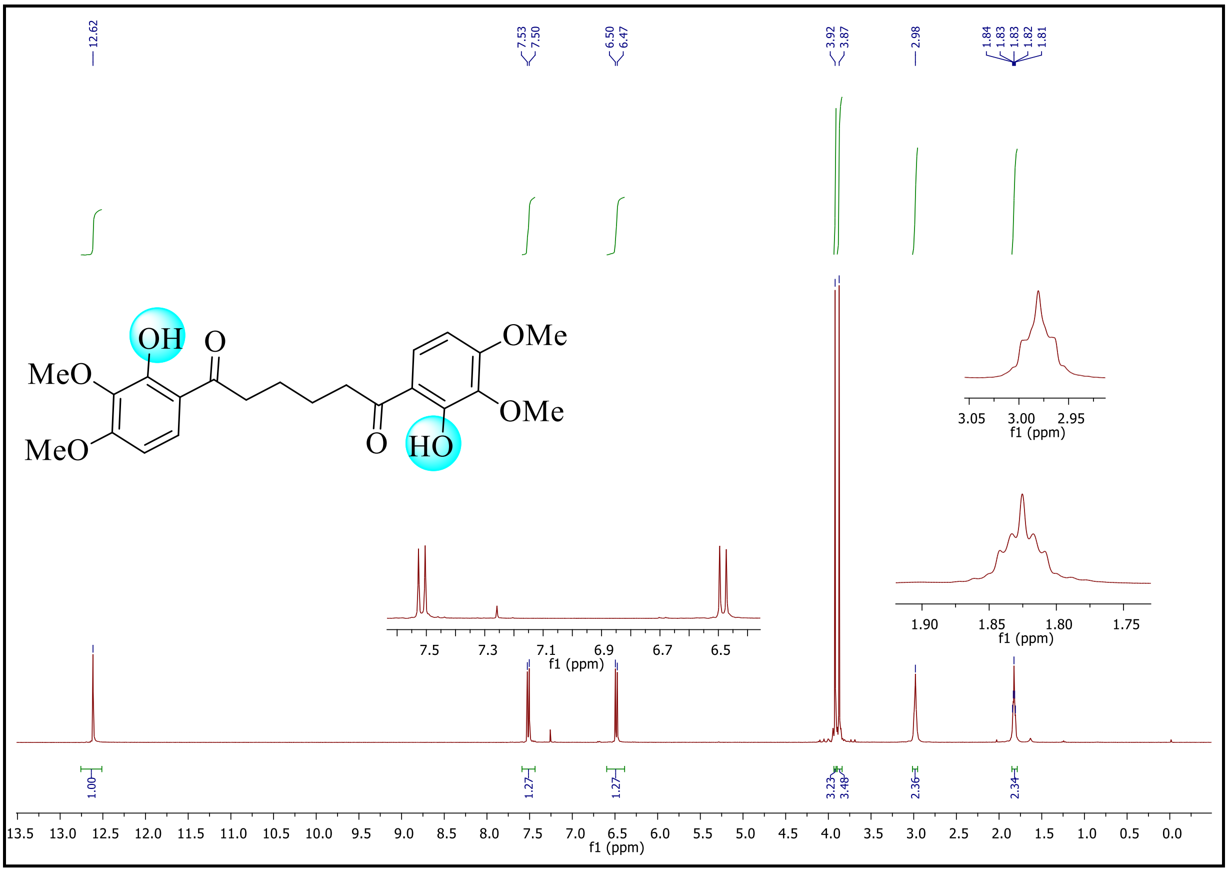

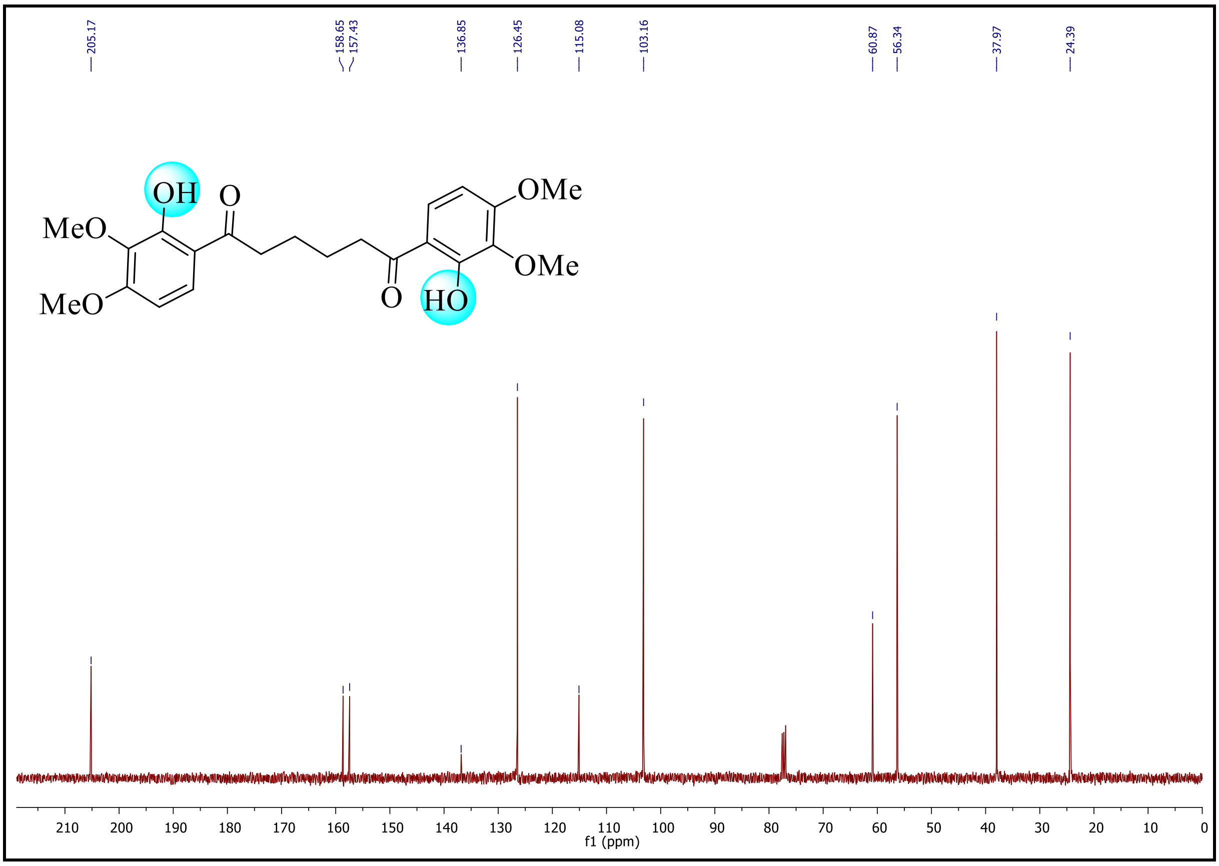

Figure S5. ^{1}H NMR and ^{13}C NMR spectra of **12**.

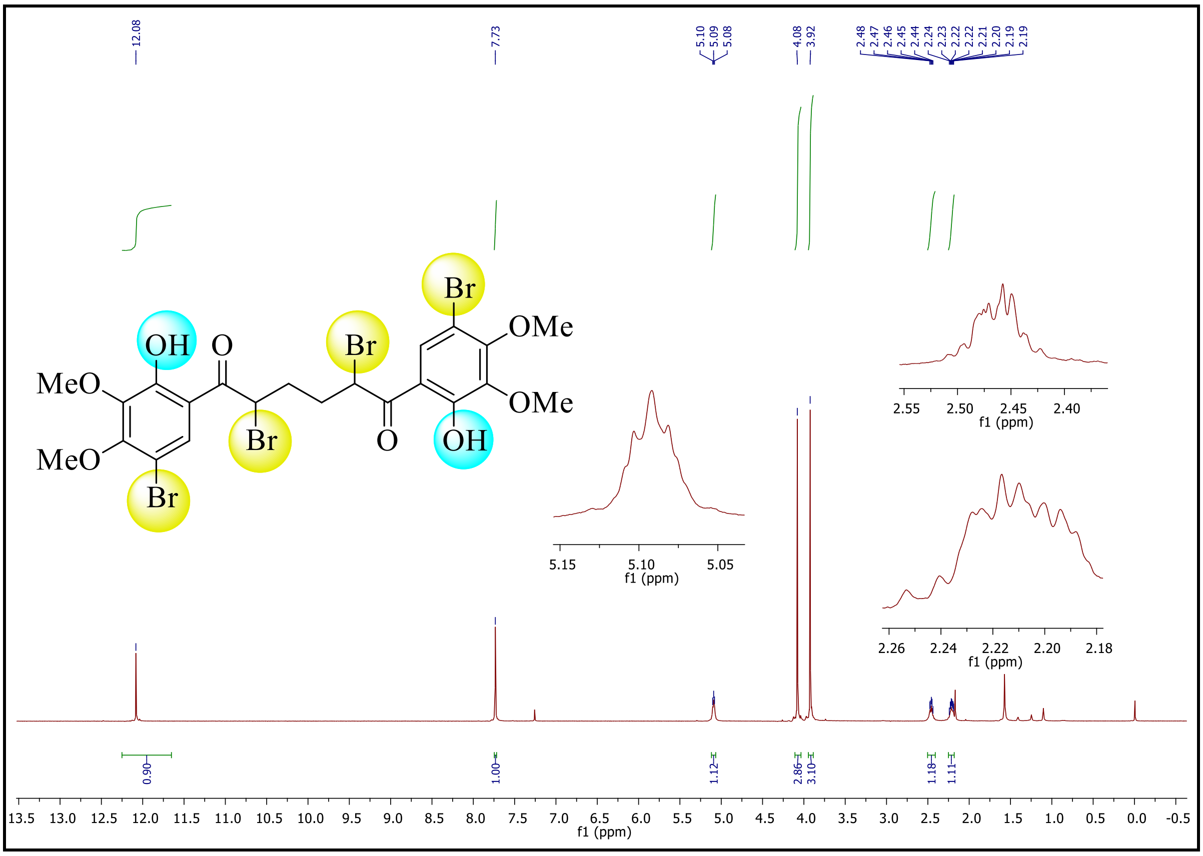

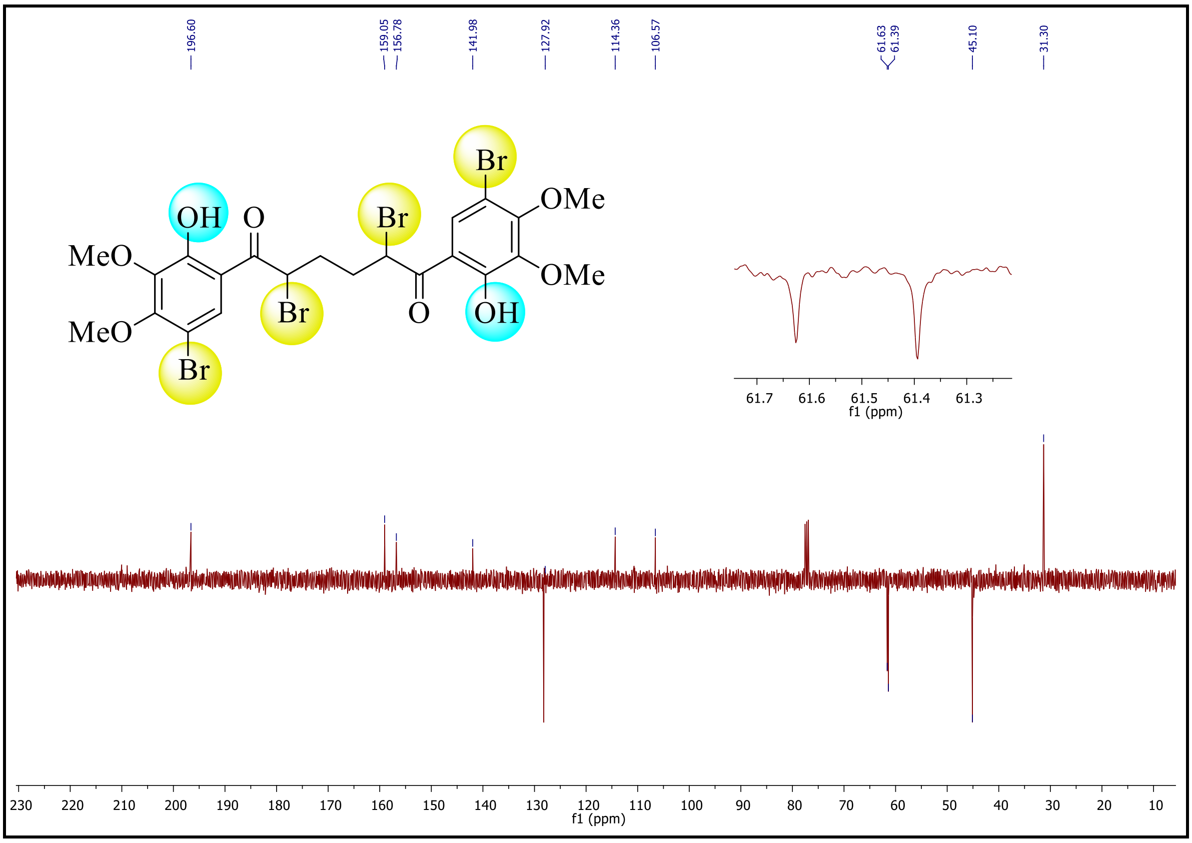

Figure S6. ^{1}H NMR and ^{13}C NMR spectra of **13**.

Table S2. ^{1}H and ^{13}C Isotropic chemical shift values of **8-13** calculated with the GIAO method by using B3LYP and M06-2X with 6-311+G(d,p) basis set.

8 (B3LYP/6-311+G (d, p))

Atoms (H)	Shift (ppm)
7 H	6,39
8 H	6,68
14 H	4,12
16 H	2,35
17 H	2,50
19 H	2,59
20 H	7,64
25 H	7,51
28 H	6,65
36 H	4,02
37 H	3,80
38 H	3,96
40 H	4,99
41 H	3,37
42 H	4,03
44 H	4,30
45 H	3,72
46 H	3,74
48 H	3,56
49 H	4,73
50 H	4,01
52 H	4,18
53 H	3,69
54 H	4,07
56 H	4,16
57 H	3,72
58 H	3,76
59 H	2,19

Atoms(C)	Shift (ppm)
1 C	109,82
2 C	129,32
3 C	138,39
4 C	160,43
5 C	152,34
6 C	164,12
9 C	220,64
11 C	73,75
12 C	32,49
13 C	146,32
15 C	39,78
18 C	145,54
21 C	131,54

22 C	131,01
23 C	163,30
24 C	111,20
26 C	152,96
27 C	162,43
35 C	66,56
39 C	62,55
43 C	57,33
47 C	61,72
51 C	62,91
55 C	56,91

8 (M06-2X/6-311+G(d,p))

Atoms(H)	Shift (ppm)
7 H	6,83
8 H	7,08
14 H	3,95
16 H	2,11
17 H	2,12
19 H	2,46
20 H	7,95
25 H	8,07
28 H	6,90
36 H	3,64
37 H	3,84
38 H	3,99
40 H	4,90
41 H	3,10
42 H	4,42
44 H	4,20
45 H	3,47
46 H	3,50
48 H	3,31
49 H	4,06
50 H	3,98
52 H	3,82
53 H	3,56
54 H	4,32
56 H	4,09
57 H	3,31
58 H	3,56
59 H	1,45

Atoms(C)	Shift (ppm)
1 C	118,33
2 C	140,10
3 C	146,71
4 C	166,60
5 C	158,08
6 C	171,30
9 C	235,07
11 C	70,87
12 C	27,59
13 C	156,57
15 C	35,71
18 C	158,42
21 C	138,69
22 C	141,17
23 C	171,78
24 C	119,75
26 C	156,65
27 C	168,40
35 C	62,43
39 C	57,85
43 C	52,04
47 C	57,90
51 C	59,47
55 C	52,51

9 (B3LYP/6-311+G (d, p))

Atoms(H)	Shift (ppm)
7 H	6,55
8 H	7,50
15 H	2,86
16 H	2,68
18 H	2,19
23 H	7,21
26 H	6,48
34 H	5,08
35 H	3,58
36 H	4,19
38 H	4,98
39 H	3,34
40 H	3,97
42 H	4,40
43 H	3,86
44 H	3,94
46 H	3,56
47 H	5,23
48 H	4,00

50 H	4,41
51 H	3,60
52 H	3,96
54 H	4,20
55 H	3,70
56 H	3,74
57 H	2,08
58 H	2,62
59 H	3,55

Atoms(C)	Shift (ppm)
1 C	109,60
2 C	133,21
3 C	136,86
4 C	162,81
5 C	148,60
6 C	167,24
9 C	202,38
11 C	153,11
12 C	41,94
13 C	161,40
14 C	30,99
17 C	44,92
19 C	134,37
20 C	131,86
21 C	161,18
22 C	109,72
24 C	152,59
25 C	162,92
33 C	63,86
37 C	62,15
41 C	58,86
45 C	63,13
49 C	62,59
53 C	56,70

9 (M06-2X/6-311+G(d,p))

Atoms(H)	Shift (ppm)
7 H	7,28
8 H	8,37
15 H	2,77
16 H	2,68
18 H	2,21
23 H	7,27
26 H	6,46
34 H	4,65
35 H	3,13
36 H	3,99
38 H	4,59
39 H	3,15

40 H	3,96
42 H	4,50
43 H	3,61
44 H	3,73
46 H	3,26
47 H	4,56
48 H	3,83
50 H	3,65
51 H	3,22
52 H	4,37
54 H	4,34
55 H	3,25
56 H	3,32
57 H	2,16
58 H	2,49
59 H	3,51

Atoms(C)	Shift (ppm)
1 C	118,91
2 C	143,64
3 C	141,39
4 C	173,36
5 C	156,96
6 C	175,52
9 C	215,81
11 C	169,30
12 C	37,54
13 C	159,93
14 C	28,68
17 C	39,06
19 C	142,05
20 C	142,90
21 C	168,50
22 C	119,38
24 C	157,29
25 C	168,95
33 C	62,13
37 C	57,85
41 C	51,25
45 C	59,25
49 C	58,46
53 C	49,70

10 (B3LYP/6-311+G (d, p))

Atoms(H)	Shift (ppm)
7 H	8,19
8 H	6,91
13 H	3,41

14 H	3,09
16 H	1,57
17 H	1,85
19 H	1,57
20 H	1,85
21 H	3,09
22 H	3,41
30 H	8,19
32 H	6,91
40 H	4,28
41 H	3,90
42 H	3,96
44 H	4,25
45 H	3,63
46 H	4,04
48 H	3,63
49 H	4,25
50 H	4,04
52 H	3,90
53 H	4,28
54 H	3,96
56 H	3,53
57 H	4,98
58 H	4,10
60 H	4,98
61 H	3,53
62 H	4,10

Atoms(C)	Shift (ppm)
1 C	150,99
2 C	166,18
3 C	131,56
4 C	133,91
5 C	110,95
6 C	168,07
9 C	207,09
11 C	51,81
12 C	29,94
15 C	29,94
18 C	51,81
23 C	207,09
25 C	131,56
26 C	166,17
27 C	133,91
28 C	150,99
29 C	110,95
31 C	168,07
39 C	58,45
43 C	62,59
47 C	62,59
51 C	58,45
55 C	62,66
59 C	62,66

10 (M06-2X/6-311+G(d,p))

Atoms(H)	Shift (ppm)
7 H	8,47
8 H	7,00
13 H	3,60
14 H	3,01
16 H	1,88
17 H	1,47
19 H	1,88
20 H	1,47
21 H	3,01
22 H	3,60
30 H	8,47
32 H	7,00
40 H	4,49
41 H	3,63
42 H	3,84
44 H	3,57
45 H	3,03
46 H	4,14
48 H	3,03
49 H	3,57
50 H	4,15
52 H	3,63
53 H	4,49
54 H	3,84
56 H	3,34
57 H	4,81
58 H	4,30
60 H	4,81
61 H	3,34
62 H	4,31

Atoms(C)	Shift (ppm)
1 C	152,93
2 C	174,72
3 C	138,18
4 C	143,00
5 C	118,35
6 C	174,33
9 C	220,98
11 C	50,67
12 C	23,77
15 C	23,77
18 C	50,67
23 C	220,98

25 C	138,18
26 C	174,72
27 C	143,00
28 C	152,92
29 C	118,35
31 C	174,33
39 C	51,73
43 C	58,84
47 C	58,84
51 C	51,73
55 C	62,38
59 C	62,38

11 (B3LYP/6-311+G (d, p))

Atoms(H)	Shift (ppm)
7 H	8,36
8 H	6,83
13 H	3,52
14 H	3,03
16 H	1,64
17 H	1,76
19 H	1,72
20 H	1,78
21 H	3,18
22 H	3,23
30 H	7,79
32 H	6,65
35 H	12,95
41 H	4,37
42 H	3,91
43 H	4,00
45 H	4,24
46 H	3,57
47 H	3,99
49 H	4,52
50 H	3,45
51 H	3,74
53 H	3,87
54 H	4,34
55 H	3,96
57 H	3,51
58 H	5,08
59 H	4,15

Atoms(C)	Shift (ppm)
1 C	151,49
2 C	165,64
3 C	130,90
4 C	134,16

5 C	111,40
6 C	167,60
9 C	206,56
11 C	51,08
12 C	29,39
15 C	28,28
18 C	44,19
23 C	214,53
25 C	121,28
26 C	167,83
27 C	132,91
28 C	145,02
29 C	106,70
31 C	168,71
40 C	59,36
44 C	62,56
48 C	61,59
52 C	59,60
56 C	62,88

11 (M06-2X/6-311+G(d,p))

Atoms(H)	Shift (ppm)
7 H	8,89
8 H	6,74
13 H	3,47
14 H	2,93
16 H	1,45
17 H	1,52
19 H	1,64
20 H	1,66
21 H	3,12
22 H	3,17
30 H	8,26
32 H	6,68
35 H	12,84
41 H	4,22
42 H	3,39
43 H	3,53
45 H	3,77
46 H	3,47
47 H	4,10
49 H	3,98
50 H	3,24
51 H	3,94
53 H	3,50
54 H	4,50
55 H	3,58
57 H	3,30
58 H	4,90
59 H	4,04

Atoms(C)	Shift (ppm)
1 C	157,90
2 C	174,01
3 C	138,48
4 C	144,00
5 C	120,70
6 C	176,15
9 C	219,00
11 C	49,34
12 C	23,36
15 C	22,73
18 C	41,78
23 C	229,07
25 C	128,54
26 C	173,18
27 C	143,75
28 C	149,71
29 C	114,37
31 C	174,39
40 C	52,59
44 C	59,24
48 C	58,68
52 C	51,51
56 C	59,13

12 (B3LYP/6-311+G (d, p))

Atoms(H)	Shift (ppm)
7 H	7,71
8 H	6,63
13 H	3,15
14 H	3,19
16 H	1,73
17 H	1,69
19 H	1,73
20 H	1,69
21 H	3,19
22 H	3,15
30 H	7,71
32 H	6,63
34 H	12,89
36 H	12,89
42 H	4,29
43 H	3,83
44 H	3,92
46 H	3,49
47 H	4,57
48 H	3,88

50 H	4,57
51 H	3,49
52 H	3,88
54 H	3,83
55 H	4,29
56 H	3,92

Atoms(C)	Shift (ppm)
1 C	144,42
2 C	167,99
3 C	120,50
4 C	133,61
5 C	107,01
6 C	168,88
9 C	213,92
11 C	44,05
12 C	28,11
15 C	28,11
18 C	44,05
23 C	213,92
25 C	120,50
26 C	167,99
27 C	133,61
28 C	144,42
29 C	107,01
31 C	168,88
41 C	59,49
45 C	61,46
49 C	61,46
53 C	59,49

12 (M06-2X/6-311+G(d,p))

Atoms(H)	Shift (ppm)
7 H	8,29
8 H	6,60
13 H	3,01
14 H	2,99
16 H	1,55
17 H	1,58
19 H	1,55
20 H	1,58
21 H	2,99
22 H	3,01
30 H	8,29
32 H	6,60

34 H	12,60
36 H	12,60
42 H	4,51
43 H	3,52
44 H	3,64
46 H	3,30
47 H	3,77
48 H	4,06
50 H	3,77
51 H	3,30
52 H	4,06
54 H	3,52
55 H	4,51
56 H	3,64

Atoms(C)	Shift (ppm)
1 C	147,91
2 C	173,35
3 C	127,52
4 C	144,29
5 C	114,15
6 C	173,85
9 C	228,00
11 C	41,02
12 C	21,96
15 C	21,96
18 C	41,02
23 C	228,00
25 C	127,52
26 C	173,35
27 C	144,29
28 C	147,91
29 C	114,15
31 C	173,86
41 C	52,22
45 C	58,40
49 C	58,40
53 C	52,22

13 (B3LYP/6-311+G (d, p))

Atoms(H)	Shift (ppm)
7 H	7,83
12 H	5,28
14 H	2,40
15 H	2,45
17 H	2,40
18 H	2,45
19 H	5,28
27 H	7,83

30 H	12,77
32 H	12,77
38 H	4,30
39 H	4,94
40 H	3,96
42 H	3,43
43 H	4,75
44 H	3,99
46 H	4,75
47 H	3,43
48 H	3,99
50 H	4,94
51 H	4,30
52 H	3,96

Atoms(C)	Shift (ppm)
1 C	148,49
2 C	170,11
3 C	117,65
4 C	136,34
5 C	129,28
6 C	167,82
8 C	206,45
10 C	66,54
11 C	35,88
13 C	35,88
16 C	66,54
20 C	206,45
22 C	117,65
23 C	170,11
24 C	136,34
25 C	148,49
26 C	129,28
28 C	167,82
37 C	66,65
41 C	61,92
45 C	61,92
49 C	66,65

13 (M06-2X/6-311+G(d,p))

Atoms(H)	Shift (ppm)
7 H	7,59
12 H	4,05
14 H	1,82
15 H	1,71
17 H	1,82
18 H	1,71
19 H	4,05

27 H	7,59
30 H	12,04
32 H	12,04
38 H	4,62
39 H	4,66
40 H	3,71
42 H	3,22
43 H	3,76
44 H	3,61
46 H	3,76
47 H	3,22
48 H	3,61
50 H	4,66
51 H	4,62
52 H	3,71

Atoms(C)	Shift (ppm)
1 C	155,77
2 C	176,64
3 C	125,25
4 C	147,09
5 C	130,91
6 C	175,83
8 C	219,53
10 C	58,93
11 C	30,80
13 C	30,80
16 C	58,93
20 C	219,53
22 C	125,25
23 C	176,64
24 C	147,09
25 C	155,77
26 C	130,91
28 C	175,83
37 C	59,96
41 C	57,73
45 C	57,73
49 C	59,96

Table S3. Charges accumulated on all atoms by NBO analysis of **8-13**.

8 (B3LYP/6-311+G (d, p))

Atom	No	Natural Charge
C	1	-0.29084
C	2	-0.17005

C	3	-0.19001
C	4	0.31303
C	5	0.23876
C	6	0.29869
H	7	0.21408
H	8	0.22147
C	9	0.61010
O	10	-0.55870
C	11	-0.31864
C	12	-0.36854
C	13	-0.08642
H	14	0.22554
C	15	-0.41473
H	16	0.20601
H	17	0.22064
C	18	-0.14754
H	19	0.20436
H	20	0.22269
C	21	-0.00386
C	22	-0.21911
C	23	0.27910
C	24	-0.28179
H	25	0.22011
C	26	0.23436
C	27	0.29689
H	28	0.21817
O	29	-0.57219
O	30	-0.55972
O	31	-0.54385
O	32	-0.58127
O	33	-0.57430
O	34	-0.53743
C	35	-0.19572
H	36	0.16530
H	37	0.18446
H	38	0.18362
C	39	-0.20196
H	40	0.18531
H	41	0.16195
H	42	0.18290
C	43	-0.20700
H	44	0.19200
H	45	0.17432
H	46	0.17099
C	47	-0.20343
H	48	0.16836
H	49	0.18828
H	50	0.18058
C	51	-0.19685
H	52	0.17510
H	53	0.16762
H	54	0.18191
C	55	-0.20738
H	56	0.19170
H	57	0.17314
H	58	0.17057

H	59	0.21135

8 M06-2X/6-311+G(d,p)

Atom	No	Natural Charge
C	1	-0.29956
C	2	-0.17443
C	3	-0.20092
C	4	0.31418
C	5	0.24127
C	6	0.30621
H	7	0.22182
H	8	0.22956
C	9	0.62520
O	10	-0.55797
C	11	-0.33125
C	12	-0.38073
C	13	-0.08528
H	14	0.23230
C	15	-0.42664
H	16	0.21304
H	17	0.22852
C	18	-0.15325
H	19	0.21122
H	20	0.22884
C	21	-0.01648
C	22	-0.23636
C	23	0.28179
C	24	-0.25237
H	25	0.22503
C	26	0.23763
C	27	0.29475
H	28	0.22061
O	29	-0.58219
O	30	-0.56877
O	31	-0.55567
O	32	-0.59157
O	33	-0.57960
O	34	-0.54872
C	35	-0.19821
H	36	0.16630
H	37	0.18737
H	38	0.18722
C	39	-0.20661
H	40	0.18805
H	41	0.16572
H	42	0.18537
C	43	-0.20702
H	44	0.19416

H	45	0.17564
H	46	0.17189
C	47	-0.20636
H	48	0.17308
H	49	0.18803
H	50	0.18387
C	51	-0.19810
H	52	0.17516
H	53	0.17152
H	54	0.18451
C	55	-0.20865
H	56	0.19404
H	57	0.17405
H	58	0.17183
H	59	0.21986

9 (B3LYP/6-311+G (d, p))

Atom	No	Natural Charge
C	1	-0.30154
C	2	-0.13883
C	3	-0.18664
C	4	0.31871
C	5	0.21496
C	6	0.31196
H	7	0.21466
H	8	0.22804
C	9	0.54056
O	10	-0.55002
C	11	-0.09885
C	12	-0.39248
C	13	0.00944
C	14	-0.37073
H	15	0.21724
H	16	0.19924
C	17	-0.40081
H	18	0.19546
C	19	0.00915
C	20	-0.19768
C	21	0.27872
C	22	-0.28230
H	23	0.20549
C	24	0.23152
C	25	0.29516
H	26	0.20927
O	27	-0.56080
O	28	-0.56753
O	29	-0.54196
O	30	-0.57038
O	31	-0.57771
O	32	-0.53998
C	33	-0.19806

H	34	0.18428
H	35	0.16609
H	36	0.18372
C	37	-0.20251
H	38	0.18811
H	39	0.16118
H	40	0.18248
C	41	-0.20726
H	42	0.19251
H	43	0.17583
H	44	0.17238
C	45	-0.20477
H	46	0.16222
H	47	0.19150
H	48	0.18034
C	49	-0.19835
H	50	0.17627
H	51	0.17175
H	52	0.17986
C	53	-0.20672
H	54	0.19066
H	55	0.17025
H	56	0.17053
H	57	0.19892
H	58	0.20187
H	59	0.21734

=============================

9 M06-2X/6-311+G(d,p)

Atom	No	Natural Charge
C	1	-0.30876
C	2	-0.13753
C	3	-0.21295
C	4	0.33235
C	5	0.21789
C	6	0.32566
H	7	0.22213
H	8	0.23890
C	9	0.56106
O	10	-0.54578
C	11	-0.13279
C	12	-0.40608
C	13	0.10070
C	14	-0.38049
H	15	0.22081
H	16	0.20978
C	17	-0.43777
H	18	0.20146
C	19	-0.01542
C	20	-0.21101
C	21	0.27237
C	22	-0.30042
H	23	0.20889

C	24	0.23383
C	25	0.30502
H	26	0.21817
O	27	-0.57744
O	28	-0.57428
O	29	-0.55187
O	30	-0.58229
O	31	-0.58483
O	32	-0.54971
C	33	-0.20196
H	34	0.18560
H	35	0.16865
H	36	0.19076
C	37	-0.20508
H	38	0.18937
H	39	0.16225
H	40	0.18679
C	41	-0.20771
H	42	0.19546
H	43	0.17770
H	44	0.17324
C	45	-0.20731
H	46	0.16444
H	47	0.19322
H	48	0.18412
C	49	-0.19935
H	50	0.17685
H	51	0.17501
H	52	0.18299
C	53	-0.20713
H	54	0.19340
H	55	0.17128
H	56	0.17172
H	57	0.20521
H	58	0.20390
H	59	0.22146

============================

10 (B3LYP/6-311+G (d, p))

Atom	No	Natural Charge
C	1	0.22199
C	2	0.32681
C	3	-0.20877
C	4	-0.11794
C	5	-0.30972
C	6	0.32612
H	7	0.23313
H	8	0.21471
C	9	0.57283
O	10	-0.58153
C	11	-0.46579
C	12	-0.38122

H	13	0.22970
H	14	0.22219
C	15	-0.38130
H	16	0.19860
H	17	0.20339
C	18	-0.46564
H	19	0.19858
H	20	0.20337
H	21	0.22218
H	22	0.22967
C	23	0.57103
O	24	-0.57938
C	25	-0.20842
C	26	0.32679
C	27	-0.11798
C	28	0.22195
C	29	-0.30998
H	30	0.23299
C	31	0.32611
H	32	0.21471
O	33	-0.57718
O	34	-0.57718
O	35	-0.57566
O	36	-0.53307
O	37	-0.57567
O	38	-0.53309
C	39	-0.20821
H	40	0.19372
H	41	0.17491
H	42	0.17250
C	43	-0.19757
H	44	0.17556
H	45	0.16976
H	46	0.18324
C	47	-0.19757
H	48	0.16976
H	49	0.17556
H	50	0.18324
C	51	-0.20821
H	52	0.17491
H	53	0.19372
H	54	0.17250
C	55	-0.20514
H	56	0.16452
H	57	0.19215
H	58	0.18601
C	59	-0.20515
H	60	0.19215
H	61	0.16452
H	62	0.18600

===========================

10 M06-2X/6-311+G(d,p)

Atom	No	Natural Charge

C	1	0.21089
C	2	0.33926
C	3	-0.21329
C	4	-0.13578
C	5	-0.31662
C	6	0.33565
H	7	0.23842
H	8	0.22195
C	9	0.59168
O	10	-0.57871
C	11	-0.48038
C	12	-0.39301
H	13	0.24241
H	14	0.21873
C	15	-0.39301
H	16	0.20977
H	17	0.20442
C	18	-0.48038
H	19	0.20977
H	20	0.20442
H	21	0.21873
H	22	0.24242
C	23	0.59168
O	24	-0.57871
C	25	-0.21329
C	26	0.33926
C	27	-0.13578
C	28	0.21089
C	29	-0.31662
H	30	0.23842
C	31	0.33565
H	32	0.22195
O	33	-0.58310
O	34	-0.58310
O	35	-0.58323
O	36	-0.54539
O	37	-0.58323
O	38	-0.54539
C	39	-0.20825
H	40	0.19553
H	41	0.17531
H	42	0.17328
C	43	-0.20183
H	44	0.17840
H	45	0.17192
H	46	0.18564
C	47	-0.20184
H	48	0.17191
H	49	0.17841
H	50	0.18564
C	51	-0.20824
H	52	0.17531
H	53	0.19553
H	54	0.17328
C	55	-0.20007

H	56	0.18097
H	57	0.18432
H	58	0.18116
C	59	-0.20007
H	60	0.18431
H	61	0.18097
H	62	0.18116

============================

11 (B3LYP/6-311+G (d, p))

Atom	No	Natural Charge
C	1	0.22190
C	2	0.32467
C	3	-0.20165
C	4	-0.11864
C	5	-0.31005
C	6	0.32717
H	7	0.23356
H	8	0.21513
C	9	0.57079
O	10	-0.58255
C	11	-0.46697
C	12	-0.38227
H	13	0.23392
H	14	0.21953
C	15	-0.38036
H	16	0.20133
H	17	0.20264
C	18	-0.46301
H	19	0.20239
H	20	0.20420
H	21	0.22102
H	22	0.22065
C	23	0.56997
O	24	-0.62734
C	25	-0.23863
C	26	0.35789
C	27	-0.15710
C	28	0.21607
C	29	-0.30161
H	30	0.21027
C	31	0.33466
H	32	0.21348
O	33	-0.57803
O	34	-0.67409
H	35	0.50256
O	36	-0.57580
O	37	-0.53284
O	38	-0.55832
O	39	-0.51804
C	40	-0.20839
H	41	0.19425
H	42	0.17525
H	43	0.17283
C	44	-0.19778

```
      H   45    0.17629
      H   46    0.16962
      H   47    0.18372
      C   48   -0.19915
      H   49    0.18188
      H   50    0.16239
      H   51    0.18314
      C   52   -0.20913
      H   53    0.17367
      H   54    0.19516
      H   55    0.17134
      C   56   -0.20554
      H   57    0.16412
      H   58    0.19366
      H   59    0.18619
 ============================
```

11 M06-2X/6-311+G(d,p)

```
                 Natural
   Atom  No    Charge
 --------------------------
      C    1    0.22305
      C    2    0.32866
      C    3   -0.21360
      C    4   -0.12352
      C    5   -0.31853
      C    6    0.33534
      H    7    0.23886
      H    8    0.22227
      C    9    0.58706
      O   10   -0.57747
      C   11   -0.48215
      C   12   -0.39356
      H   13    0.24083
      H   14    0.22330
      C   15   -0.39227
      H   16    0.20800
      H   17    0.20849
      C   18   -0.47963
      H   19    0.20889
      H   20    0.21044
      H   21    0.22699
      H   22    0.22668
      C   23    0.59373
      O   24   -0.62736
      C   25   -0.25417
      C   26    0.36714
      C   27   -0.15825
      C   28    0.21472
      C   29   -0.31469
      H   30    0.21600
      C   31    0.34304
      H   32    0.22067
      O   33   -0.59014
      O   34   -0.69010
```

H	35	0.51663
O	36	-0.58273
O	37	-0.54420
O	38	-0.56670
O	39	-0.53332
C	40	-0.20882
H	41	0.19639
H	42	0.17576
H	43	0.17353
C	44	-0.19903
H	45	0.17637
H	46	0.17314
H	47	0.18621
C	48	-0.19997
H	49	0.18050
H	50	0.16547
H	51	0.18560
C	52	-0.20926
H	53	0.17439
H	54	0.19693
H	55	0.17214
C	56	-0.20954
H	57	0.16620
H	58	0.19609
H	59	0.18963

============================

12 (B3LYP/6-311+G (d, p))

Atom	No	Natural Charge
C	1	0.21658
C	2	0.35853
C	3	-0.23954
C	4	-0.15703
C	5	-0.30083
C	6	0.33581
H	7	0.20975
H	8	0.21393
C	9	0.56879
O	10	-0.62804
C	11	-0.46303
C	12	-0.38122
H	13	0.22169
H	14	0.22085
C	15	-0.38122
H	16	0.20473
H	17	0.20380
C	18	-0.46302
H	19	0.20472
H	20	0.20380
H	21	0.22085
H	22	0.22169
C	23	0.56879

O	24	-0.62804
C	25	-0.23954
C	26	0.35853
C	27	-0.15703
C	28	0.21658
C	29	-0.30083
H	30	0.20975
C	31	0.33581
H	32	0.21393
O	33	-0.67297
H	34	0.50272
O	35	-0.67297
H	36	0.50271
O	37	-0.55806
O	38	-0.51729
O	39	-0.55806
O	40	-0.51729
C	41	-0.20934
H	42	0.19572
H	43	0.17396
H	44	0.17165
C	45	-0.19909
H	46	0.16268
H	47	0.18160
H	48	0.18366
C	49	-0.19908
H	50	0.18160
H	51	0.16268
H	52	0.18366
C	53	-0.20934
H	54	0.17396
H	55	0.19572
H	56	0.17165

=============================

12 M06-2X/6-311+G(d,p)

Atom	No	Natural Charge
C	1	0.21476
C	2	0.36822
C	3	-0.25525
C	4	-0.15812
C	5	-0.31419
C	6	0.34398
H	7	0.21549
H	8	0.22117
C	9	0.59292
O	10	-0.62747
C	11	-0.47979
C	12	-0.39292
H	13	0.22721
H	14	0.22737
C	15	-0.39294
H	16	0.21049

H	17	0.21060
C	18	-0.47980
H	19	0.21049
H	20	0.21061
H	21	0.22737
H	22	0.22721
C	23	0.59285
O	24	-0.62755
C	25	-0.25534
C	26	0.36780
C	27	-0.15817
C	28	0.21481
C	29	-0.31419
H	30	0.21549
C	31	0.34394
H	32	0.22117
O	33	-0.68773
H	34	0.51640
O	35	-0.68684
H	36	0.51634
O	37	-0.56667
O	38	-0.53350
O	39	-0.56669
O	40	-0.53351
C	41	-0.20933
H	42	0.19749
H	43	0.17473
H	44	0.17240
C	45	-0.19938
H	46	0.16627
H	47	0.17870
H	48	0.18615
C	49	-0.19940
H	50	0.17868
H	51	0.16627
H	52	0.18615
C	53	-0.20934
H	54	0.17473
H	55	0.19749
H	56	0.17240

============================

13 (B3LYP/6-311+G (d, p))

Atom	No	Natural Charge
C	1	0.20876
C	2	0.36677
C	3	-0.22931
C	4	-0.17360
C	5	-0.15988
C	6	0.32188
H	7	0.22734
C	8	0.53748
O	9	-0.61415

C	10	-0.28133
C	11	-0.41491
H	12	0.22296
C	13	-0.41492
H	14	0.22873
H	15	0.23409
C	16	-0.28145
H	17	0.22874
H	18	0.23408
H	19	0.22296
C	20	0.53813
O	21	-0.61423
C	22	-0.23445
C	23	0.37416
C	24	-0.17323
C	25	0.20645
C	26	-0.15984
H	27	0.22725
C	28	0.32184
O	29	-0.66586
H	30	0.50466
O	31	-0.66647
H	32	0.50473
O	33	-0.57231
O	34	-0.53147
O	35	-0.57246
O	36	-0.53141
C	37	-0.19432
H	38	0.18576
H	39	0.18342
H	40	0.18281
C	41	-0.20351
H	42	0.16675
H	43	0.18940
H	44	0.18587
C	45	-0.20347
H	46	0.18937
H	47	0.16674
H	48	0.18586
C	49	-0.19432
H	50	0.18344
H	51	0.18575
H	52	0.18280
Br	53	0.08918
Br	54	0.00477
Br	55	0.08915
Br	56	0.00479

13 M06-2X/6-311+G(d,p)

Atom	No	Natural Charge
C	1	0.20389
C	2	0.38414
C	3	-0.25536

C	4	-0.17392
C	5	-0.17870
C	6	0.33033
H	7	0.23505
C	8	0.56964
O	9	-0.61158
C	10	-0.31327
C	11	-0.42544
H	12	0.23281
C	13	-0.42544
H	14	0.23297
H	15	0.23950
C	16	-0.31327
H	17	0.23297
H	18	0.23950
H	19	0.23281
C	20	0.56964
O	21	-0.61158
C	22	-0.25536
C	23	0.38414
C	24	-0.17392
C	25	0.20389
C	26	-0.17870
H	27	0.23505
C	28	0.33033
O	29	-0.68273
H	30	0.51863
O	31	-0.68273
H	32	0.51863
O	33	-0.58064
O	34	-0.54766
O	35	-0.58064
O	36	-0.54766
C	37	-0.19373
H	38	0.18685
H	39	0.18193
H	40	0.18467
C	41	-0.20513
H	42	0.16935
H	43	0.18735
H	44	0.18930
C	45	-0.20513
H	46	0.18735
H	47	0.16935
H	48	0.18930
C	49	-0.19373
H	50	0.18193
H	51	0.18685
H	52	0.18468
Br	53	0.09856
Br	54	0.02321
Br	55	0.09856
Br	56	0.02321

============================

Table S4. Cartesian coordinates for the optimized structures of **8-13**.

8 (B3LYP/6-311+G (d, p))

Atomic Number	Coordinates (Angstroms) X	Y	Z
6	-2.190909	1.920780	-0.187637
6	-1.357793	0.804583	-0.227693
6	-1.888743	-0.481885	-0.268466
6	-3.284787	-0.645082	-0.256223
6	-4.136931	0.466440	-0.207119
6	-3.576308	1.761055	-0.160592
1	-1.751166	2.908070	-0.161413
1	-0.285670	0.948080	-0.245769
6	-1.013419	-1.701331	-0.417939
8	-1.099103	-2.388921	-1.417152
6	-0.080777	-2.112204	0.729978
6	-0.915170	-2.410241	2.012899
6	0.924750	-1.054026	1.201646
1	0.422986	-3.012892	0.370594
6	-0.696643	-1.196222	2.941560
1	-0.511788	-3.313607	2.476695
1	-1.967366	-2.599207	1.792628
6	0.558683	-0.569064	2.403505
1	-0.600514	-1.488174	3.992964
1	1.086063	0.215961	2.923775
6	2.083703	-0.690564	0.364157
6	2.184241	-1.186202	-0.944950
6	3.142123	0.144902	0.803722
6	3.247671	-0.878459	-1.789644
1	1.412173	-1.837435	-1.331144
6	4.210993	0.470338	-0.037109
6	4.271633	-0.045726	-1.345780
1	3.264160	-1.296028	-2.787002
8	-3.796886	-1.912753	-0.167052
8	-5.482566	0.246813	-0.095079
8	-4.464608	2.792139	-0.060931
8	3.117491	0.693379	2.062666
8	5.233211	1.246201	0.450276
8	5.354832	0.326584	-2.083060
6	-4.402362	-2.446105	-1.351990
1	-5.256356	-1.840221	-1.664390
1	-3.661966	-2.513292	-2.153436
1	-4.748268	-3.444802	-1.087309
6	-6.334561	0.799347	-1.107177
1	-6.374970	1.886942	-1.043716
1	-5.993504	0.500769	-2.104798
1	-7.322704	0.378755	-0.922094
6	-3.959828	4.116932	0.058615
1	-4.834570	4.758925	0.149085
1	-3.335874	4.225682	0.951545
1	-3.385813	4.407630	-0.827470
6	4.054074	0.133618	2.996938
1	3.851296	-0.931828	3.146795

1	5.079501	0.277515	2.652498
1	3.901180	0.667547	3.934848
6	5.222913	2.611498	0.015793
1	5.318295	2.675448	-1.071082
1	4.305656	3.110858	0.344115
1	6.084295	3.086161	0.485305
6	5.489239	-0.201118	-3.397273
1	6.421589	0.205833	-3.785250
1	5.548274	-1.294315	-3.382454
1	4.659998	0.111250	-4.040971
1	-1.534650	-0.488744	2.894769

8 (M06-2X/6-311+G (d, p))

Atomic Number	Coordinates (Angstroms) X	Y	Z
6	-2.109517	1.819606	-0.581350
6	-1.273651	0.715239	-0.439646
6	-1.804154	-0.548407	-0.220712
6	-3.191765	-0.707058	-0.143328
6	-4.043484	0.387406	-0.287174
6	-3.490534	1.664316	-0.490656
1	-1.673823	2.794947	-0.747809
1	-0.200720	0.846672	-0.511842
6	-0.946958	-1.780227	-0.104412
8	-0.996807	-2.644324	-0.947228
6	-0.093962	-1.934008	1.150467
6	-1.009443	-1.897986	2.401463
6	0.867424	-0.773154	1.387064
1	0.423674	-2.890501	1.048363
6	-0.886455	-0.465804	2.953561
1	-0.615754	-2.610621	3.128692
1	-2.038026	-2.183570	2.171722
6	0.416927	0.013096	2.376681
1	-0.903861	-0.434820	4.045844
1	0.893760	0.929958	2.691496
6	2.048947	-0.609127	0.520520
6	2.212491	-1.428617	-0.599781
6	3.050763	0.356110	0.750940
6	3.297178	-1.308943	-1.463851
1	1.474033	-2.188556	-0.821636
6	4.153361	0.471446	-0.088826
6	4.277484	-0.355026	-1.216583
1	3.363126	-1.967150	-2.319060
8	-3.700849	-1.937692	0.153702
8	-5.384233	0.194291	-0.150091
8	-4.383134	2.684011	-0.565189
8	2.964587	1.222750	1.805092
8	5.132855	1.373058	0.213214
8	5.372133	-0.139690	-1.984643
6	-4.262459	-2.624983	-0.961402
1	-5.107814	-2.066056	-1.371000

1	-3.496424	-2.781417	-1.725131
1	-4.611813	-3.584971	-0.585740
6	-6.180340	0.517438	-1.287092
1	-6.284132	1.596382	-1.401501
1	-5.739269	0.093010	-2.195086
1	-7.155304	0.064961	-1.111546
6	-3.872673	3.997780	-0.676879
1	-4.738458	4.656061	-0.678373
1	-3.229194	4.241740	0.173624
1	-3.314274	4.127177	-1.608942
6	3.760578	0.828288	2.921533
1	3.428913	-0.146153	3.293814
1	4.814888	0.787796	2.639217
1	3.612406	1.584190	3.691463
6	5.019274	2.594113	-0.510266
1	5.072740	2.406921	-1.585857
1	4.078845	3.093901	-0.259856
1	5.859496	3.215524	-0.205138
6	5.548489	-0.972466	-3.113111
1	6.479588	-0.655006	-3.577033
1	5.627067	-2.023053	-2.817716
1	4.725592	-0.853352	-3.824730
1	-1.706794	0.172549	2.601940

9 (B3LYP/6-311+G (d, p))

Atomic Number	Coordinates (Angstroms) X	Y	Z
6	-4.175555	-0.610641	-2.046242
6	-2.824293	-0.292106	-2.057525
6	-2.149483	0.097968	-0.900643
6	-2.859270	0.117377	0.319218
6	-4.223559	-0.214495	0.352670
6	-4.885104	-0.557393	-0.845341
1	-4.670006	-0.881796	-2.968536
1	-2.261746	-0.340054	-2.981512
6	-0.682872	0.415207	-1.037147
8	-0.027712	-0.192677	-1.871233
6	-0.087699	1.525662	-0.242424
6	-0.807695	2.850702	-0.053302
6	1.167264	1.567069	0.251648
6	0.070280	3.581643	0.983692
1	-1.840526	2.736830	0.279004
1	-0.835013	3.392079	-1.009089
6	1.464904	2.938609	0.837240
1	-0.320808	3.383724	1.985909
6	2.135735	0.452563	0.305815
6	1.761394	-0.785726	0.831104
6	3.461430	0.597395	-0.149551
6	2.646114	-1.856928	0.908756
1	0.750384	-0.909939	1.199104
6	4.353074	-0.482183	-0.116896

6	3.948773	-1.713203	0.432593
1	2.312341	-2.792409	1.336345
8	-2.179570	0.512038	1.435053
8	-4.907549	-0.079207	1.534331
8	-6.221647	-0.796278	-0.735340
8	3.832443	1.787290	-0.725806
8	5.630870	-0.318179	-0.594597
8	4.894646	-2.697210	0.443642
6	-2.286133	-0.242449	2.646984
1	-3.168097	0.046640	3.217681
1	-2.315605	-1.315803	2.434284
1	-1.383777	-0.014407	3.215097
6	-5.537857	-1.249189	2.074294
1	-6.355036	-1.588323	1.437928
1	-4.808369	-2.056631	2.202129
1	-5.926630	-0.954377	3.048834
6	-6.962971	-1.088407	-1.915657
1	-7.995011	-1.212033	-1.591851
1	-6.903775	-0.265475	-2.634684
1	-6.615642	-2.013710	-2.386313
6	4.905180	2.504409	-0.098630
1	4.642043	2.757494	0.934247
1	5.830318	1.927932	-0.115468
1	5.026036	3.421737	-0.674834
6	5.883860	-0.926820	-1.868896
1	5.784821	-2.013123	-1.807450
1	5.200445	-0.529580	-2.625703
1	6.910160	-0.668020	-2.129286
6	4.543803	-3.961594	0.988759
1	5.435847	-4.579993	0.901277
1	4.264237	-3.876983	2.044431
1	3.723341	-4.425093	0.430240
1	0.084286	4.664860	0.842618
1	2.009539	2.877825	1.784329
1	2.098487	3.497607	0.139754

9 (M06-2X/6-311+G (d, p))

Atomic Number	Coordinates (Angstroms) X	Y	Z
6	-4.064913	-0.960275	-1.798669
6	-2.809392	-0.445899	-2.079574
6	-2.079969	0.270812	-1.134929
6	-2.638693	0.457669	0.142934
6	-3.893610	-0.073508	0.451519
6	-4.616002	-0.770678	-0.530988
1	-4.608546	-1.495286	-2.564608
1	-2.359192	-0.597771	-3.053079
6	-0.704161	0.731489	-1.531641
8	-0.175046	0.254862	-2.512224
6	-0.031822	1.800181	-0.738170
6	-0.609210	3.194626	-0.651820

6	1.138114	1.696666	-0.097094
6	0.197655	3.810669	0.503995
1	-1.685045	3.193460	-0.464611
1	-0.434395	3.720658	-1.598325
6	1.526505	3.033531	0.510553
1	-0.338121	3.617606	1.437209
6	1.914661	0.455616	0.078321
6	1.277455	-0.747367	0.373514
6	3.312708	0.441305	-0.043739
6	1.978906	-1.936620	0.538423
1	0.198472	-0.752606	0.486555
6	4.031460	-0.743645	0.101523
6	3.363859	-1.941068	0.399088
1	1.440854	-2.845218	0.770477
8	-1.962612	1.200035	1.056480
8	-4.436578	0.189626	1.675680
8	-5.845587	-1.197414	-0.160346
8	3.965235	1.590925	-0.389981
8	5.389633	-0.735447	-0.049260
8	4.154183	-3.038324	0.514964
6	-1.679910	0.621243	2.325361
1	-2.462881	0.863956	3.044668
1	-1.579160	-0.464957	2.239875
1	-0.724643	1.041413	2.643886
6	-4.652152	-0.942413	2.513390
1	-5.387376	-1.619571	2.078741
1	-3.711230	-1.475815	2.683250
1	-5.024142	-0.552526	3.459644
6	-6.656819	-1.812397	-1.144097
1	-7.601997	-2.034557	-0.654297
1	-6.830745	-1.135384	-1.985115
1	-6.204858	-2.740644	-1.505432
6	4.844089	2.108790	0.605896
1	4.288279	2.325503	1.523575
1	5.651793	1.407140	0.817080
1	5.249171	3.035292	0.201384
6	5.811381	-1.195137	-1.331296
1	5.488142	-2.226441	-1.492072
1	5.407393	-0.546889	-2.113963
1	6.899017	-1.146779	-1.335252
6	3.524436	-4.262491	0.830163
1	4.319056	-5.002884	0.889962
1	3.009467	-4.203205	1.794162
1	2.811095	-4.552876	0.052322
1	0.332688	4.888854	0.405812
1	1.948662	2.924817	1.513330
1	2.286951	3.514002	-0.113642

----

10 (B3LYP/6-311+G (d, p))

Atomic Number	Coordinates (Angstroms) X	Y	Z
6	-6.441620	0.563469	-0.070958
6	-5.050852	0.421390	-0.178191
6	-4.457429	-0.860809	-0.189305
6	-5.310221	-1.969284	-0.110006
6	-6.687659	-1.846281	-0.021459
6	-7.267198	-0.573789	-0.002292
1	-4.851917	-2.949964	-0.121269
1	-7.300883	-2.735195	0.034474
6	-2.981553	-1.162985	-0.260302
8	-2.623998	-2.323143	-0.382757
6	-1.944930	-0.055816	-0.152788
6	-0.508518	-0.570560	-0.058114
1	-2.196467	0.580598	0.701246
1	-2.058618	0.600567	-1.023437
6	0.508490	0.570431	0.058391
1	-0.421812	-1.239512	0.805171
1	-0.284680	-1.188591	-0.933078
6	1.944908	0.055702	0.153043
1	0.421767	1.239388	-0.804890
1	0.284660	1.188456	0.933361
1	2.058624	-0.600661	1.023706
1	2.196435	-0.580725	-0.700981
6	2.981517	1.162892	0.260537
8	2.623943	2.323034	0.383086
6	4.457393	0.860769	0.189366
6	5.050873	-0.421408	0.178316
6	5.310124	1.969274	0.109851
6	6.441633	-0.563432	0.070923
6	6.687558	1.846326	0.021147
1	4.851776	2.949935	0.121078
6	7.267151	0.573857	0.002041
1	7.300734	2.735261	-0.034954
8	-4.274446	1.548767	-0.214677
8	4.274505	-1.548800	0.215054
8	-6.992390	1.820230	-0.088999
8	-8.599948	-0.331707	0.090548
8	6.992475	-1.820163	0.089006
8	8.599898	0.331818	-0.090952
6	-9.493441	-1.440331	0.133939
1	-10.492049	-1.010698	0.188992
1	-9.409983	-2.052266	-0.769548
1	-9.314543	-2.061995	1.017067
6	-7.431936	2.313952	1.184252
1	-8.225876	1.684137	1.592672
1	-6.593230	2.366033	1.885490
1	-7.819003	3.316202	1.002623
6	7.431926	-2.313940	-1.184258
1	6.593153	-2.366133	-1.885407
1	8.225775	-1.684089	-1.592800
1	7.819094	-3.316146	-1.002599
6	9.493338	1.440468	-0.134755

1	9.410080	2.052559	0.768646
1	10.491949	1.010862	-0.189974
1	9.314201	2.061971	-1.017947
6	-4.372844	2.357532	-1.398515
1	-4.078702	1.777152	-2.279748
1	-5.384526	2.743744	-1.526237
1	-3.673091	3.180192	-1.255697
6	4.373176	-2.357500	1.398914
1	5.384890	-2.743688	1.526443
1	4.079213	-1.777073	2.280177
1	3.673401	-3.180175	1.256292

10 (M06-2X/6-311+G (d, p))

Atomic Number	Coordinates (Angstroms) X	Y	Z
6	-6.374829	-0.544836	0.078891
6	-5.003370	-0.414659	-0.139388
6	-4.417489	0.859733	-0.235842
6	-5.247152	1.973482	-0.118663
6	-6.609561	1.862026	0.112927
6	-7.182130	0.593679	0.212993
1	-4.787643	2.950539	-0.204631
1	-7.211341	2.754692	0.210699
6	-2.950048	1.124414	-0.433008
8	-2.590268	2.234159	-0.759870
6	-1.931273	0.033219	-0.170164
6	-0.502588	0.557591	-0.137414
1	-2.051245	-0.732857	-0.942941
1	-2.190449	-0.466917	0.769761
6	0.502569	-0.557786	0.136832
1	-0.277695	1.046378	-1.089837
1	-0.416945	1.336640	0.627196
6	1.931243	-0.033390	0.169662
1	0.277646	-1.046604	1.089231
1	0.416964	-1.336812	-0.627806
1	2.190442	0.466816	-0.770219
1	2.051166	0.732635	0.942496
6	2.950035	-1.124577	0.432478
8	2.590263	-2.234384	0.759138
6	4.417489	-0.859805	0.235526
6	5.003341	0.414629	0.139424
6	5.247211	-1.973499	0.118229
6	6.374823	0.544902	-0.078639
6	6.609648	-1.861944	-0.113152
1	4.787728	-2.950592	0.203938
6	7.182184	-0.593555	-0.212874
1	7.211476	-2.754568	-0.211020
8	-4.240452	-1.521764	-0.334463
8	4.240377	1.521671	0.334661
8	-6.932691	-1.789828	0.158987
8	-8.496626	0.351573	0.409794

8	6.932642	1.789935	-0.158407
8	8.496700	-0.351358	-0.409431
6	-9.354559	1.466760	0.559680
1	-10.350343	1.057794	0.713729
1	-9.068172	2.068041	1.427289
1	-9.349860	2.091501	-0.338364
6	-7.530748	-2.214965	-1.062723
1	-8.320140	-1.521899	-1.363830
1	-6.773808	-2.286486	-1.850038
1	-7.960429	-3.197351	-0.873217
6	7.530515	2.214850	1.063469
1	6.773463	2.286180	1.850695
1	8.319895	1.521757	1.364544
1	7.960178	3.197292	0.874221
6	9.354692	-1.466479	-0.559473
1	9.068484	-2.067522	-1.427307
1	10.350493	-1.057442	-0.713221
1	9.349842	-2.091474	0.338393
6	-4.297816	-2.562715	0.642768
1	-4.650317	-2.174844	1.600338
1	-4.963914	-3.359839	0.314854
1	-3.280283	-2.941840	0.747292
6	4.297884	2.562862	-0.642305
1	4.963918	3.359914	-0.314085
1	4.650551	2.175230	-1.599912
1	3.280363	2.941998	-0.746904

11 (B3LYP/6-311+G (d, p))

Atomic Number	Coordinates (Angstroms) X	Y	Z
6	6.290952	0.608294	0.017826
6	4.913460	0.349739	0.061650
6	4.429636	-0.977615	0.019126
6	5.374951	-2.009696	-0.045495
6	6.739766	-1.771623	-0.060307
6	7.211064	-0.455537	-0.026941
1	4.999786	-3.024497	-0.080877
1	7.427231	-2.605274	-0.100604
6	2.983591	-1.402694	0.017876
8	2.717553	-2.592612	-0.032324
6	1.860185	-0.378926	0.072730
6	0.465229	-1.000092	0.005125
1	2.010462	0.347913	-0.731339
1	1.974977	0.204238	0.993671
6	-0.640785	0.057693	0.087744
1	0.369600	-1.572694	-0.923486
1	0.352056	-1.728662	0.814410
6	-2.038206	-0.559504	0.019102
1	-0.546238	0.629211	1.016664
1	-0.528418	0.786071	-0.721453
1	-2.155253	-1.129280	-0.910936

1	-2.169768	-1.288543	0.827774
6	-3.168289	0.455408	0.100575
8	-2.896458	1.659870	0.197014
6	-4.564511	0.001896	0.062667
6	-5.617181	0.959417	0.139872
6	-4.913146	-1.356015	-0.033254
6	-6.958573	0.547268	0.105825
6	-6.230554	-1.772753	-0.059469
1	-4.134053	-2.105088	-0.089175
6	-7.263509	-0.819687	0.005400
1	-6.456787	-2.826689	-0.135196
8	4.043388	1.406992	0.076495
8	-5.377371	2.273697	0.249397
1	-4.392361	2.377683	0.261624
8	6.733045	1.905926	0.071839
8	8.521187	-0.101995	-0.046883
8	-7.974542	1.447260	0.254946
8	-8.580208	-1.127414	-0.022120
6	9.507452	-1.130333	-0.057621
1	10.466987	-0.616575	-0.049904
1	9.427560	-1.765036	0.830266
1	9.430981	-1.746649	-0.959039
6	7.204339	2.445063	-1.171711
1	8.070783	1.887036	-1.534074
1	6.406841	2.432695	-1.921166
1	7.494601	3.474498	-0.963906
6	-8.222559	2.307008	-0.865272
1	-7.355614	2.936664	-1.076198
1	-8.485938	1.717993	-1.750302
1	-9.069113	2.931243	-0.581261
6	-8.967606	-2.496103	-0.082917
1	-8.602281	-3.052087	0.786218
1	-10.056008	-2.491450	-0.077428
1	-8.610623	-2.972314	-1.001853
6	4.036323	2.221349	1.261119
1	3.775807	1.615839	2.135927
1	5.003702	2.700051	1.413789
1	3.266153	2.974562	1.099030

----

11 (M06-2X/6-311+G (d, p))

Atomic Number	Coordinates (Angstroms) X	Y	Z
6	-6.237084	0.625013	-0.030958
6	-4.871337	0.338927	-0.036965
6	-4.419402	-0.991798	-0.005680
6	-5.379330	-2.004259	0.026514
6	-6.739045	-1.738097	0.024056
6	-7.178089	-0.413558	-0.001167
1	-5.025416	-3.027329	0.054366
1	-7.444737	-2.556823	0.046369
6	-2.977832	-1.426140	0.013766
8	-2.719053	-2.608511	0.071100

6	-1.859759	-0.404556	-0.037381
6	-0.476942	-1.038761	0.009832
1	-2.000429	0.306979	0.781430
1	-1.980754	0.190740	-0.949584
6	0.620610	0.018281	-0.074929
1	-0.376899	-1.617801	0.933186
1	-0.376135	-1.758189	-0.808377
6	2.012905	-0.595025	-0.011235
1	0.523563	0.588496	-1.003749
1	0.509871	0.744742	0.735407
1	2.145034	-1.151600	0.923895
1	2.148438	-1.324639	-0.817699
6	3.117628	0.438334	-0.108488
8	2.827599	1.625688	-0.209785
6	4.524598	0.002714	-0.077608
6	5.559257	0.965459	-0.174187
6	4.881324	-1.345795	0.037179
6	6.898160	0.562663	-0.150245
6	6.200395	-1.753969	0.065977
1	4.106191	-2.098375	0.110549
6	7.218674	-0.792141	-0.020626
1	6.434397	-2.804371	0.159941
8	-3.995506	1.385395	-0.025208
8	5.325017	2.276094	-0.289055
1	4.352497	2.398355	-0.296822
8	-6.653405	1.924265	-0.071555
8	-8.471732	-0.027490	0.013897
8	7.892506	1.478471	-0.295973
8	8.534153	-1.077669	0.014884
6	-9.461863	-1.038795	0.015393
1	-10.417360	-0.519837	0.008713
1	-9.381830	-1.667518	-0.876051
1	-9.389382	-1.660902	0.912374
6	-7.016228	2.441232	1.206661
1	-7.841460	1.864441	1.631377
1	-6.154119	2.420032	1.879360
1	-7.333614	3.469978	1.045841
6	8.164260	2.224774	0.886090
1	7.282210	2.790023	1.195791
1	8.486806	1.556204	1.690221
1	8.971354	2.911453	0.637512
6	8.916927	-2.435430	0.118265
1	8.552759	-3.013410	-0.736112
1	10.004178	-2.437535	0.119993
1	8.549028	-2.879722	1.047906
6	-3.927295	2.118628	-1.248552
1	-3.644031	1.453326	-2.070851
1	-4.884269	2.593423	-1.467755
1	-3.155719	2.873716	-1.107950

----

12 (B3LYP/6-311+G (d, p))

Atomic Number	Coordinates (Angstroms) X	Y	Z
6	6.924780	-0.480019	-0.175621
6	5.618991	-0.994463	-0.198979
6	4.496406	-0.121573	-0.102295
6	4.740641	1.258755	0.000848
6	6.021918	1.775580	0.017875
6	7.124699	0.905521	-0.065004
1	3.907218	1.945669	0.069960
1	6.167459	2.843110	0.100151
6	3.140078	-0.680787	-0.126049
8	2.957669	-1.901716	-0.225551
6	1.935992	0.244904	-0.027810
6	0.591988	-0.484255	-0.046921
1	1.987866	0.964732	-0.853649
1	2.030708	0.840435	0.888043
6	-0.591982	0.484195	0.046822
1	0.519891	-1.081837	-0.961058
1	0.559508	-1.200621	0.780085
6	-1.935985	-0.244965	0.027712
1	-0.519885	1.081777	0.960958
1	-0.559503	1.200560	-0.780185
1	-2.030703	-0.840493	-0.888143
1	-1.987855	-0.964796	0.853549
6	-3.140072	0.680727	0.125953
8	-2.957676	1.901656	0.225463
6	-4.496402	0.121523	0.102248
6	-5.618951	0.994447	0.198975
6	-4.740680	-1.258797	-0.000888
6	-6.924758	0.480047	0.175662
6	-6.021975	-1.775580	-0.017871
1	-3.907281	-1.945736	-0.070030
6	-7.124726	-0.905486	0.065048
1	-6.167553	-2.843105	-0.100145
8	5.480888	-2.322497	-0.316452
1	4.507906	-2.504665	-0.316666
8	-5.480781	2.322474	0.316455
1	-4.507791	2.504596	0.316648
8	8.004795	-1.298044	-0.343220
8	8.413421	1.313261	-0.046399
8	-8.004739	1.298111	0.343305
8	-8.413462	-1.313184	0.046486
6	8.696354	2.707285	0.022120
1	9.781832	2.785169	0.007511
1	8.282250	3.239853	-0.839695
1	8.312628	3.148146	0.947839
6	8.334498	-2.140416	0.769275
1	8.568480	-1.536052	1.652127
1	7.520035	-2.832971	0.991702
1	9.219835	-2.699564	0.468622

6	-8.334479	2.140467	-0.769190
1	-7.519987	2.832958	-0.991711
1	-8.568579	1.536088	-1.652001
1	-9.219750	2.699688	-0.468481
6	-8.696442	-2.707198	-0.022033
1	-8.282328	-3.239786	0.839765
1	-9.781922	-2.785047	-0.007390
1	-8.312759	-3.148066	-0.947768

12 (M06-2X/6-311+G (d, p))

Atomic Number	Coordinates (Angstroms) X	Y	Z
6	6.885606	-0.490525	-0.211525
6	5.588072	-1.011266	-0.210994
6	4.474039	-0.142593	-0.103847
6	4.712467	1.232717	0.003339
6	5.990305	1.756061	0.016148
6	7.087669	0.887217	-0.084638
1	3.875928	1.915470	0.082536
1	6.133267	2.823032	0.106857
6	3.110576	-0.698886	-0.111818
8	2.920009	-1.907185	-0.198114
6	1.923923	0.239651	-0.011173
6	0.586039	-0.486199	-0.049861
1	1.989334	0.967218	-0.828017
1	2.019313	0.816861	0.915790
6	-0.586037	0.486216	0.049839
1	0.518962	-1.067641	-0.974192
1	0.546857	-1.212752	0.767029
6	-1.923920	-0.239638	0.011172
1	-0.518955	1.067669	0.974164
1	-0.546863	1.212761	-0.767059
1	-2.019309	-0.816874	-0.915777
1	-1.989327	-0.967183	0.828035
6	-3.110575	0.698898	0.111788
8	-2.920015	1.907204	0.198009
6	-4.474037	0.142600	0.103847
6	-5.588071	1.011274	0.210963
6	-4.712461	-1.232715	-0.003281
6	-6.885604	0.490530	0.211522
6	-5.990298	-1.756063	-0.016065
1	-3.875921	-1.915469	-0.082456
6	-7.087664	-0.887218	0.084691
1	-6.133257	-2.823038	-0.106730
8	5.469890	-2.338186	-0.313282
1	4.513467	-2.548836	-0.302047
8	-5.469893	2.338199	0.313197
1	-4.513470	2.548850	0.301958
8	7.952653	-1.317309	-0.374474
8	8.373475	1.285017	-0.066347

8	-7.952651	1.317319	0.374442
8	-8.373470	-1.285021	0.066421
6	8.640441	2.671940	0.018656
1	9.723182	2.767788	-0.005623
1	8.207466	3.207849	-0.830929
1	8.256970	3.091197	0.953588
6	8.345943	-1.985776	0.819727
1	8.645732	-1.257896	1.579899
1	7.532071	-2.611040	1.194982
1	9.197345	-2.610848	0.556991
6	-8.345969	1.985707	-0.819794
1	-7.532110	2.610955	-1.195103
1	-8.645765	1.257775	-1.579914
1	-9.197372	2.610788	-0.557083
6	-8.640433	-2.671947	-0.018539
1	-8.207454	-3.207829	0.831061
1	-9.723174	-2.767796	0.005748
1	-8.256965	-3.091231	-0.953460

12 (B3LYP/6-311+G (d, p))

Atomic Number	Coordinates (Angstroms) X	Y	Z
6	-6.717273	-1.449753	0.090759
6	-5.363961	-1.726136	0.367337
6	-4.391250	-0.691929	0.302010
6	-4.825530	0.613035	-0.012868
6	-6.151637	0.885042	-0.244243
6	-7.130452	-0.137752	-0.183463
1	-4.121508	1.431664	-0.043310
6	-2.990343	-1.022109	0.561456
8	-2.640094	-2.172502	0.848467
6	-1.903038	0.047390	0.452489
6	-0.538966	-0.531568	0.109065
1	-2.180077	0.833533	-0.244888
6	0.538963	0.531557	-0.109054
1	-0.244874	-1.244572	0.882190
1	-0.671771	-1.112750	-0.810481
6	1.903035	-0.047399	-0.452478
1	0.244871	1.244561	-0.882179
1	0.671768	1.112739	0.810492
1	2.180076	-0.833540	0.244900
6	2.990339	1.022102	-0.561450
8	2.640087	2.172493	-0.848462
6	4.391247	0.691926	-0.302006
6	5.363953	1.726136	-0.367339
6	4.825533	-0.613037	0.012874
6	6.717268	1.449759	-0.090766
6	6.151642	-0.885038	0.244243
1	4.121514	-1.431668	0.043321
6	7.130452	0.137759	0.183457
8	-5.049387	-2.991944	0.664362

1	-4.072698	-3.013609	0.824031
8	5.049372	2.991941	-0.664370
1	4.072683	3.013601	-0.824035
8	-7.646293	-2.455240	0.133686
8	-8.400260	0.218226	-0.440929
8	7.646286	2.455246	-0.133703
8	8.400264	-0.218210	0.440918
6	-9.512105	-0.398755	0.236223
1	-10.305976	0.346394	0.201920
1	-9.824927	-1.309764	-0.270775
1	-9.256493	-0.626871	1.271623
6	-7.570385	-3.420076	-0.930675
1	-7.683862	-2.927133	-1.901630
1	-6.627817	-3.967723	-0.893163
1	-8.400419	-4.106625	-0.768635
6	7.570384	3.420087	0.930656
1	6.627820	3.967738	0.893143
1	7.683861	2.927146	1.901612
1	8.400422	4.106630	0.768612
6	9.512098	0.398775	-0.236248
1	9.824917	1.309791	0.270741
1	10.305975	-0.346367	-0.201948
1	9.256474	0.626883	-1.271648
35	-6.692702	2.674622	-0.638603
35	-1.881760	0.987866	2.228361
35	6.692715	-2.674615	0.638604
35	1.881758	-0.987880	-2.228349

12 (M06-2X/6-311+G (d, p))

Atomic Number	Coordinates (Angstroms) X	Y	Z
6	-6.708492	-1.454268	0.142906
6	-5.352583	-1.735432	0.381029
6	-4.391215	-0.707108	0.287399
6	-4.824899	0.592306	-0.029995
6	-6.152420	0.864825	-0.243365
6	-7.124403	-0.155515	-0.151424
1	-4.119538	1.410698	-0.080342
6	-2.976311	-1.030465	0.506709
8	-2.609312	-2.161207	0.793529
6	-1.910520	0.052393	0.351545
6	-0.528859	-0.532988	0.129252
1	-2.170381	0.750652	-0.442356
6	0.528883	0.532944	-0.129418
1	-0.263190	-1.158780	0.984536
1	-0.605484	-1.200350	-0.735459
6	1.910542	-0.052438	-0.351717
1	0.263212	1.158738	-0.984700
1	0.605511	1.200304	0.735294
1	2.170404	-0.750700	0.442180

6	2.976334	1.030420	-0.506881
8	2.609330	2.161172	-0.793654
6	4.391228	0.707086	-0.287474
6	5.352586	1.735424	-0.381054
6	4.824911	-0.592317	0.029970
6	6.708483	1.454285	-0.142832
6	6.152422	-0.864811	0.243434
1	4.119560	-1.410718	0.080283
6	7.124395	0.155544	0.151546
8	-5.051038	-3.004982	0.659050
1	-4.085010	-3.058754	0.805575
8	5.051041	3.004963	-0.659124
1	4.085024	3.058714	-0.805732
8	-7.619567	-2.467547	0.203396
8	-8.398400	0.177509	-0.394829
8	7.619546	2.467578	-0.203276
8	8.398381	-0.177457	0.395042
6	-9.459655	-0.441640	0.339308
1	-10.253870	0.301976	0.373493
1	-9.806494	-1.343186	-0.163157
1	-9.134201	-0.692223	1.349312
6	-7.624855	-3.297004	-0.958772
1	-7.888548	-2.706788	-1.842022
1	-6.649899	-3.768139	-1.097598
1	-8.380001	-4.061656	-0.786440
6	7.624740	3.297054	0.958879
1	6.649767	3.768178	1.097628
1	7.888379	2.706858	1.842158
1	8.379887	4.061715	0.786587
6	9.459675	0.441700	-0.339031
1	9.806469	1.343256	0.163448
1	10.253903	-0.301906	-0.373155
1	9.134285	0.692268	-1.349059
35	-6.700196	2.626172	-0.656510
35	-1.979311	1.125648	2.004351
35	6.700198	-2.626143	0.656641
35	1.979322	-1.125687	-2.004527
